# Prognosis prediction and risk stratification of transarterial chemoembolization or intraarterial chemotherapy for unresectable hepatocellular carcinoma based on machine learning

**DOI:** 10.1007/s00330-024-10581-2

**Published:** 2024-01-30

**Authors:** Wendao Liu, Ran Wei, Junwei Chen, Yangyang Li, Huajin Pang, Wentao Zhang, Chao An, Chengzhi Li

**Affiliations:** 1 Department of Interventional therapy, Guangdong Provincial Hospital of Chinese Medicine and Guangdong Provincial Academy of Chinese Medical Sciences, Guangzhou, Guangdong People’s Republic of China; 2 Department of Gastrointestinal Surgery, The First Affiliated Hospital, Sun Yat-sen University, Guangzhou, Guangdong China; 3 Department of Interventional Radiology, the Third Affiliated Hospital of Sun Yat-Sen University, Guangzhou, Guangdong People’s Republic of China; 4 Department of Interventional Radiology and Vascular Surgery, The First Affiliated Hospital of Jinan University, Guangzhou, Guangdong People’s Republic of China; 5 Division of Vascular and Interventional Radiology, Department of General Surgery, Nanfang Hospital, Southern Medical University, Guangzhou, Guangdong People’s Republic of China; 6 Department of Radiology, The First Affiliated Hospital, Nanchang University, Nanchang, Jiangxi People’s Republic of China; 7 Department of Minimal invasive intervention, Sun Yat-sen University Cancer Center; State Key Laboratory of Oncology in South China; Collaborative Innovation Center for Cancer Medicine, Guangzhou, People’s Republic of China

**Keywords:** Intra-arterial therapies, Hepatocellular carcinoma, Risk scoring scale model, Machine learning, Risk stratification

## Abstract

**Objective:**

To develop and validate a risk scoring scale model (RSSM) for stratifying prognostic risk after intra-arterial therapies (IATs) for hepatocellular carcinoma (HCC).

**Methods:**

Between February 2014 and October 2022, 2338 patients with HCC who underwent initial IATs were consecutively enrolled. These patients were divided into training datasets (TD, *n* = 1700), internal validation datasets (ITD, *n* = 428), and external validation datasets (ETD, *n* = 200). Five-years death was used to predict outcome. Thirty-four clinical information were input and five supervised machine learning (ML) algorithms, including eXtreme Gradient Boosting (XGBoost), Categorical Gradient Boosting (CatBoost), Gradient Boosting Decision Tree (GBDT), Light Gradient Boosting Machine (LGBT), and Random Forest (RF), were compared using the areas under the receiver operating characteristic (AUC) with DeLong test. The variables with top important ML scores were used to build the RSSM by stepwise Cox regression.

**Results:**

The CatBoost model achieved the best discrimination when 12 top variables were input, with the AUC of 0.851 (95% confidence intervals (CI), 0.833–0.868) for TD, 0.817 (95%CI, 0.759–0.857) for ITD, and 0.791 (95%CI, 0.748–0.834) for ETD. The RSSM was developed based on the immune checkpoint inhibitors (ICI) (hazard ratios (HR), 0.678; 95%CI 0.549, 0.837), tyrosine kinase inhibitors (TKI) (HR, 0.702; 95%CI 0.605, 0.814), local therapy (HR, 0.104; 95%CI 0.014, 0.747), response to the first IAT (HR, 4.221; 95%CI 2.229, 7.994), tumor size (HR, 1.054; 95%CI 1.038, 1.070), and BCLC grade (HR, 2.375; 95%CI 1.950, 2.894). Kaplan–Meier analysis confirmed the role of RSSM in risk stratification (*p* < 0.001).

**Conclusions:**

The RSSM can stratify accurately prognostic risk for HCC patients received IAT. On the basis, an online calculator permits easy implementation of this model.

**Clinical relevance statement:**

The risk scoring scale model could be easily implemented for physicians to stratify risk and predict prognosis quickly and accurately, thereby serving as a more favorable tool to strengthen individualized intra-arterial therapies and management in patients with unresectable hepatocellular carcinoma.

**Key Points:**

*• The Categorical Gradient Boosting (CatBoost) algorithm achieved the optimal and robust predictive ability (AUC, 0.851 (95%CI, 0.833–0.868) in training datasets, 0.817 (95%CI, 0.759–0.857) in internal validation datasets, and 0.791 (95%CI, 0.748–0.834) in external validation datasets) for prediction of 5-years death of hepatocellular carcinoma (HCC) after intra-arterial therapies (IATs) among five machine learning models.*

*• We used the SHapley Additive exPlanations algorithms to explain the CatBoost model so as to resolve the black boxes of machine learning principles.*

*• A simpler restricted variable, risk scoring scale model (RSSM), derived by stepwise Cox regression for risk stratification after intra-arterial therapies for hepatocellular carcinoma* *,* *provides the potential forewarning to adopt combination strategies for high-risk patients.*

**Supplementary Information:**

The online version contains supplementary material available at 10.1007/s00330-024-10581-2.

## Introduction

Hepatocellular carcinoma (HCC) is the third most common malignant tumor and ranks as the fourth leading cause of death due cancer globally [1, 2]. Unfortunately, > 50% of patients with HCC often present with a high tumor burden and distant metastasis when they receive the initial diagnosis [3–5]. Transarterial chemoembolization (TACE) and hepatic arterial infusion chemotherapy (HAIC) are standard intra-arterial therapies (IATs), important interventional technologies for patients with unresectable HCC [5]. The National Comprehensive Cancer Network guidelines recommend TACE as the first-line treatment for intermediate-stage HCC [6]. Moreover, the Japan Society of Hepatology recommends HAIC as an important therapeutic schedule in advanced-stage HCC [7]. The application of IAT technologies administered in combination with molecular-targeted agents and immune checkpoint inhibitors (ICIs), along with conversion therapy performed for sequential surgery or ablation, has greatly improved the survival benefits of HCC patients [8–11]. However, high-level tumor heterogeneity at the histologic, genomic, and molecular levels results in a range of individualized differences in the prognosis of HCC patients.

Questions regarding how long-term survival outcomes after IAT can be accurately predicted have great clinical significance not only for the design of clinical trials but also for guiding individualized therapeutic schemes for patients with HCC. Multiple risk factors affecting the prognosis of HCC patients undergoing IAT have been identified [12–15]. An increasing number of prognostic prediction models based on the clinicalvariables have been established and applied with the purpose of improving clinical practice. However, these models are limited by the relatively few number of inclusion variables, small derivation sample sizes, varying pathologic scoring standards, and poor clinical applicability.

Machine learning (ML) is a branch of artificial intelligence that employs statistical, probabilistic, and optimization techniques to train a machine to learn. ML algorithms learn from data, identify patterns, and make decisions with minimal human intervention by automating analytical model building. In the medical field, there are many commonly used ML algorithms [16–18]. An et al reported using five ML algorithms to establish risk prediction models for the early recurrence of HCC after microwave ablation, and this was considered robust with prediction capabilities, providing guidance and assistance to physicians [19].

Our study aimed to use ML algorithms to analyze clinical data from 2338 HCC patients who underwent IAT with long-term follow-up. We built a prognostic prediction and death risk stratification system (IAT Risk Stratification System) comprising widely accepted clinical variables to assist physicians in predicting HCC prognosis quickly and accurately before IAT.

## Materials and methods

This retrospective, multi-institutional study was conducted following the principles of the 1975 Helsinki Declaration and approval for the protocol was obtained from the Institutional Review Board of The First Affiliated Hospital of Jinan University (KY-2022–119). Due to the retrospective nature of this study, the requirement for written informed consent was waived.

### Patient enrolment

**Fig. 1:**
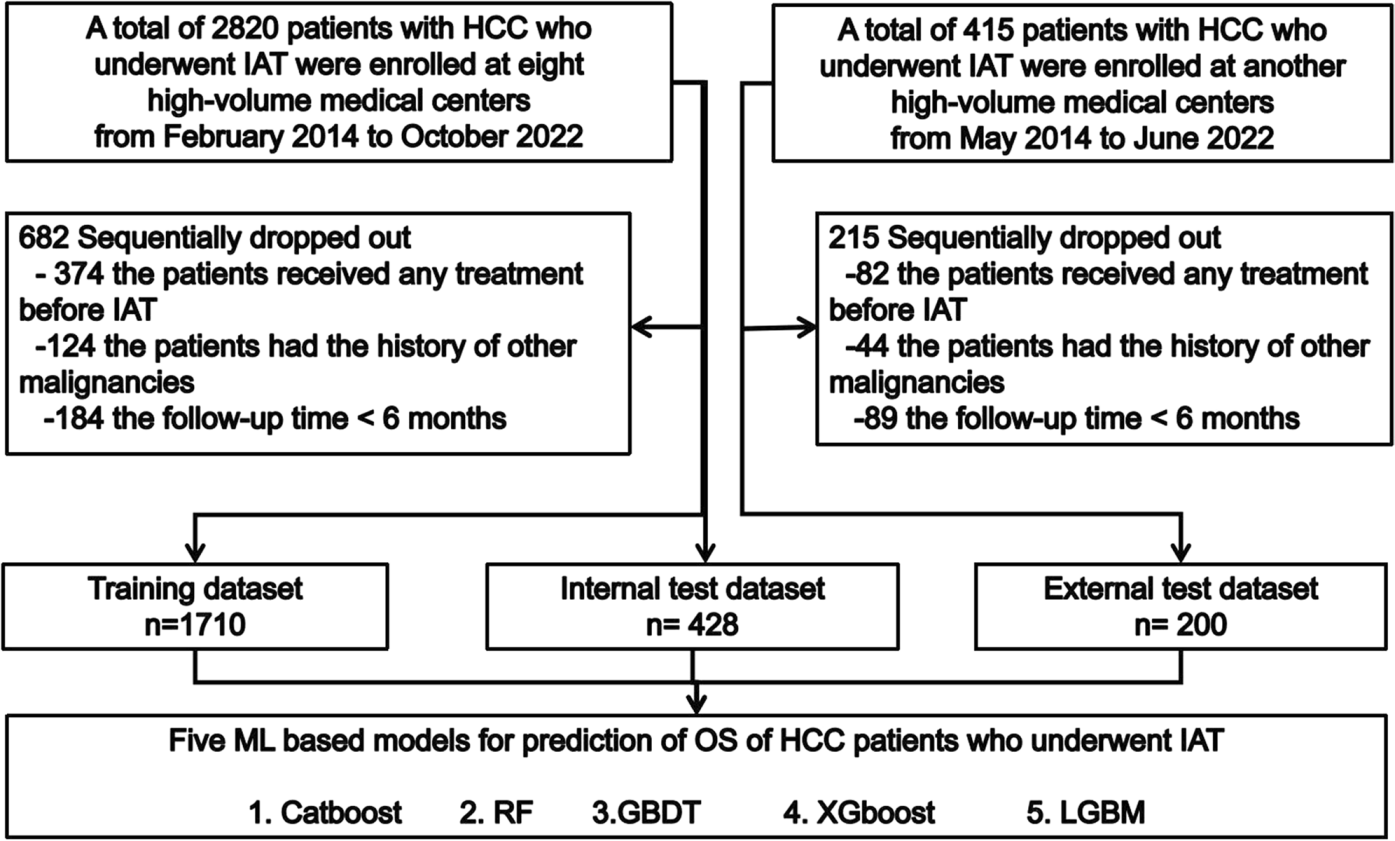
The enrolment pathway of the patients with unresectable hepatocellular carcinoma who underwent intra-arterial therapies

Between February 2014 and October 2022, a total of 2338 consecutive patients with HCC who underwent initial IATs were enrolled retrospectively from 12 medical centers in China. These clinical data were utilized with a database that was developed in-house. HCC was diagnosed based on the European Association for the Study of Liver (EASL) and the American Association for the Study of Liver Disease (AASLD) guidelines. All follow-up information was updated to March 2023. The TACE and HAIC procedures, combination scheme, and conversion therapy are described in Supplementary Information E1.1–1.3. Figure 1 demonstrates the enrolment pathways of HCC patients, and the inclusion and exclusion criteria are shown in Supplementary Information E1.4.

### Study design

The ML models were developed to predict death by estimating the risk for the oncological outcome in HCC patients who underwent initial IATs based on available clinical information. The observation window for clinical indicators was defined at the time of initial TACE or HAIC treatment. The prediction window was defined as 5 years from initial TACE or HAIC treatment. The primary endpoint of this study was 5-year death, and overall survival (OS) was defined as the period from the date of initial TACE or HAIC to the date of death or the last follow-up. For patients whose data were censored within 5 years, their outcome was defined as “nonevent” in all models. Moreover, 1-, 2-, and 3-years death predictions were also analyzed. A total of 38 variables (Table S2) closely related to clinical outcomes were collected to build the ML models**.**

### Risk scoring scale model construction

#### Datasets assignment

As shown in Fig. 1, a total of 2338 HCC patients (277 females and 2061 males; mean age, 53.6 ± 11.2 years) were enrolled in our study. Clinical data from eight medical centers were used as the primary datasets for training datasets (TD, *n* = 1700) and internal validation datasets (ITD, *n* = 428), and clinical data from four medical centers were used as the external validation datasets (ETD,* n* = 200). The abovementioned data sources are shown in Table S1**.**

#### Data collection

In this study, 38 clinical variables are collected as follows: (1) demographic and history variables (ECOG, pathology differentiation, weight, height, BMI, age, gender, comorbidities (i.e., hypertension, diabetes, heart disease, renal disease and esophageal gastric varices, etc.), etiology, CTP class, ALBI grade, ascites); (2) tumor features (maximal tumor diameter, number of tumor, tumor burden, macroscopic vascular invasion, metastasis, and BCLC stage); (3) laboratory findings (α-fetoprotein (AFP), des-γ-carboxy prothrombin (DCP), serum albumin (ALB), total bilirubin (TB), platelet counts, prothrombin time (PT), international normalized ratio (INR), aspartate aminotransferase (AST), and alanine aminotransferase (ALT)), C reactive protein (CRP), creatinine, neutrophils, lymphocyte. Albumin-bilirubin (ALBI) grades were used to replace CTP grade for their objectiveness. ALBI score was calculated before treatment using the appropriate clinical parameters and ALBI grade was defined as follows: (log 10 bilirubin [BI] [μ mol /L] × 0.66) + (albumin [AL] [g/L] ×  − 0.085), (grade 1, 2, and 3 =  ≤  − 2.60, >  − 2.60 to − 1.39, and >  − 1.39, respectively). For more detailed evaluations of patients with the middle grade of ALBI (grade 2), we used modified ALBI (mALBI) grading consisting of four levels, which included subgrading for the middle grade of 2 (2a and 2b) based on an ALBI score of –2.27 as the cutoff, which was previously reported as the value for indocyanine green retention after 15 min (ICG-R15) of 30%; (4) treatment parameters (IAT modalities, combination with TKI, combination with ICI, sequential local therapy, and the response of first IAT). The responses to IAT were assessed by dynamic contrast-enhanced CT or magnetic resonance imaging (MRI) based on modified Response Evaluation Criteria in Solid Tumor (mRECIST), including complete response (CR), partial response (PR), stable disease (SD), and progression disease (PD), which was performed every 4–6 weeks after initial IAT and evaluated independently by two radiologists (reader 1, L.Z.L., and reader 2, J.Z., with 10 years of experience) who were blinded to IAT procedures at the time of data collection.

#### ML-based model construction

Since the missing data rate exceeded 20%, four variables including pathology differentiation, weight, height, and BMI were removed from the model. Five representative supervised ML algorithms (XGBoost, CatBoost, GBDT, LGBM, and RF) were included in this study, and their parameter details are shown in Table S3. We compared the performance of five ML-based models for the prediction of 5-year death after IAT using the areas under the receiver operating characteristic (AUCs) with the DeLong test. The optimal ML-based model was used for the selection of important clinical variables and the development of a simpler restricted variable, risk scoring scale model (RSSM), derived by stepwise Cox regression for OS risk stratification. To interpret the causal relationship of the ML model, we used the Shapley Additive exPlanations (SHAP) method to explain the prediction results of each ML model [20–22]. For model interpretation, the SHAP algorithm was used to further identify the model with the best performance. The SHAP algorithm calculates the Shapley value of each variable based on game theory to determine the relative importance of each variable in the optimal performance of the ML model.

#### Candidate predictors

In the optimal ML-based model, all clinical variables were used to train and rank according to the feature’s class discrimination importance. The three least important variables were removed, and the remaining 31 feature variables were retrained in each model. Based on recursive feature elimination with cross-validation (RFECV), we performed the three least important feature elimination processes 11 times (3/6/9/12/15/18/21/24/27/30/33 features) to evaluate the risk features on the predictive performance and to select the most important features.

#### RSSM derived using stepwise Cox regression

To further expand the applicability of our predictive model by simplifying the inputs, we also constructed a stepwise Cox proportional hazards model based on the top important variables screened from the optimal model (Table S4). We used a stepwise Cox regression method because it can achieve local optimum goodness of fit while confirming the statistical significance of the predictive variables. The stepwise Cox model was not used on all 34 variables, instead targeted the top variables selected by the ML model. At the same time, risk scores were derived from variable coefficients in the Cox regression model. Finally, we assessed the discrimination ability of the applied using the AUC. The OS risk stratification of the RSSM was evaluated using Kaplan‒Meier analysis. The RSSM was incorporated into a risk stratification system for IAT for HCC patients, which is available online.

### Statistical analysis

All statistical analyses were undertaken using R software version 3.6.3 (http://www.r-project.org/). The quantitative variables with mean ± standard deviation or median with interquartile range (IQR) were compared using the Kruskal‒Wallis test. Qualitative variables described by frequency were compared using the χ^2^ test. Survival curves were calculated using the Kaplan‒Meier method and compared using the log-rank test. Univariate and multivariate Cox regression analyses were applied to calculate the logarithm of hazard ratios (HRs) and the corresponding 95% confidence intervals (CIs) of variables and to identify independent significant risk factors. The predictive parameters including AUC, sensitivity (SEN), specificity (SPE), positive predictive value (PPV), and negative predictive value (NPV) were also calculated to assess model performance.

All tests of significance were two-sided, and results with a *p* value < 0.05 were considered statistically significant.

## Results

### Baseline characteristics

**Fig. 2:**
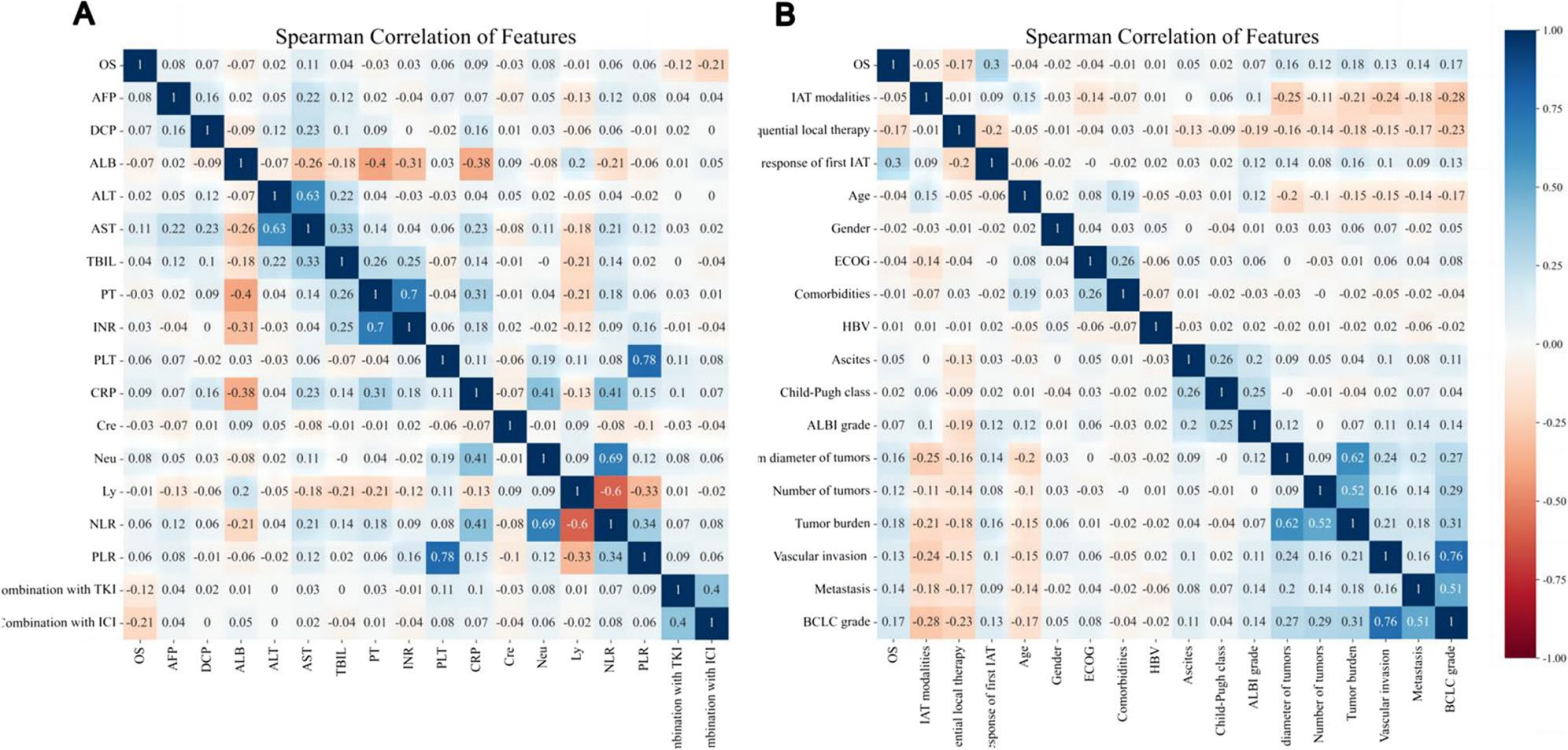
The correlation coefficient matrix heat map of all 34 variables. **A** The correlation analysis of 17 variables including AFP; **B** the correlation analysis of the other 17 variables including BCLC stage

The median follow-up period was 31.7 months (IQR, 11.6–55.5 months) in this study. In total, 1169 patients (1169/2338, 50.0%) died after receiving IATs until the final follow-up endpoint (31 March 2023). Among them, 848 (848/1700, 49.9%) deaths occurred in the TD, 224 (224/428, 52.3%) deaths occurred in the ITD, and 97 (97/200, 48.5%) deaths occurred in the ETD. Table 1 shows a comparison of baseline characteristics between the three datasets. All pre-IAT variable characteristics demonstrated superior balance and consistency (all, *p* > 0.05). Notably, the classification distributions (complete response (CR), partial response (PR), stable disease (SD), and progression disease (PD)) of the first response to IAT (e.g., first response was defined as 4–6 weeks after IATs) were also consistent across the three datasets [23]. Correlation analyses with 34 risk factors produced a correlation coefficient matrix heatmap of features (Fig. 2), which showed that the top five features that negatively correlated with 5-years survival were as follows: combination with ICI, local therapy, combination with TKI, ALB, and IAT modalities. The top five characteristics that correlated positively with 5-years survival were the following: the response of first IAT, tumor burden, BCLC grade, maximum diameter of tumor, and metastasis.

**Table 1:** The baseline characteristics of patients in three datasets

Variables	TC (*n* = 1710)	ITC (*n* = 428)	ETC (*n* = 200)	*p* value
Demographic and history (*n* = 8)				
Age (years)^a^	53.4 ± 11.9	54.8 ± 12.6	53.2 ± 13.2	0.077
Gender^b^				0.463
Male	1499 (87.66%)	384 (89.72%)	178 (89%)	
Female	211 (12.34%)	44 (10.28%)	22 (11%)	
ECOG^b^				0.940
PS 0	1557 (91.05%)	391 (91.36%)	181 (90.5%)	
PS 1	153 (8.95%)	37 (8.64%)	19 (9.5%)	
Comorbidities^b^				0.089
Absence	1503 (87.89%)	364 (85.05%)	182 (91.0%)	
Presence	207 (12.11%)	64 (14.95%)	18 (9.0%)	
HBV^b^				0.548
Absence	137 (8.01%)	41 (9.58%)	18 (9.0%)	
Presence	1573 (91.99%)	387 (90.42%)	182 (91.0%)	
Ascites^b^				0.169
Absence	1493 (87.31%)	385 (89.95%)	170 (85.0%)	
Presence	217 (12.69%)	43 (10.05%)	30 (15.0%)	
Child–Pugh class^b^				0.827
A	1668 (97.54%)	416 (97.2%)	196 (98.0%)	
B	42 (2.46%)	12 (2.8%)	4 (2.0%)	
ALBI grade^b^				0.641
1	876 (51.23%)	222 (51.87%)	103 (51.5%)	
2a	383 (22.4%)	103 (24.07%)	50 (25.0%)	
2b	412 (24.09%)	95 (22.2%)	46 (23.0%)	
3	39 (2.28%)	8 (1.87%)	1 (0.5%)	
Tumor characteristics (*n* = 6)				
Maximum diameter of tumors (cm)^c^	9.5	9.5	9.6	0.975
Number of tumors^b^				0.621
1–3	867 (50.7%)	206 (48.13%)	102 (51.0%)	
> 3	843 (49.3%)	222 (51.87%)	98 (49.0%)	
Tumor burden^b^				0.160
< 6	229 (13.39%)	70 (16.36%)	29 (14.5%)	
6–12	297 (17.37%)	55 (12.85%)	32 (16.0%)	
> 12	1184 (69.24%)	303 (70.79%)	139 (69.5%)	
Vascular invasion^b^				0.336
Absence	932 (54.5%)	243 (56.78%)	101 (50.5%)	
Presence	778 (45.5%)	185 (43.22%)	99 (49.5%)	
Metastasis^b^				0.183
Absence	1266 (74.04%)	301 (70.33%)	140 (70.0%)	
Presence	444 (25.96%)	127 (29.67%)	60 (30.0%)	
BCLC grade^b^				0.598
A	329 (19.24%)	79 (18.46%)	38 (19.0%)	
B	417 (24.39%)	102 (23.83%)	39 (19.5%)	
C	964 (56.37%)	247 (57.71%)	123 (61.5%)	
Laboratory findings (*n* = 15)				
AFP (µg/mL)^b^				0.670
< 400	801 (46.84%)	210 (49.07%)	92 (46.0%)	
> 400	909 (53.16%)	218 (50.93%)	108 (54.0%)	
DCP (µg/mL)^b^				0.039
< 400	301 (17.6%)	96 (22.43%)	31 (15.5%)	
> 400	1409 (82.4%)	332 (77.57%)	169 (84.5%)	
ALT (U/L)^c^	62.6	65.3	74.7	0.191
AST(U/L)^c^	86.0	87.7	90.2	0.865
ALB (g/L)^a^	39.5 ± 5.5	39.6 ± 5.0	39.5 ± 4.5	0.948
TBIL (μmol/L)^c^	18.6	19.2	21.0	0.281
PT(s)^a^	12.6 ± 1.4	12.6 ± 1.3	12.6 ± 1.4	0.979
INR^a^	1.09 ± 0.13	1.10 ± 0.11	1.09 ± 0.16	0.167
PLT (× 10^9^)^c^	191	195	208	0.100
Cre (μmol/L)^c^	71.3	71.6	70.8	0.888
CRP (μmol/L)^c^	21.1	22.7	22.8	0.653
Ly^a^	1.5 ± 0.6	1.6 ± 0.6	1.4 ± 0.6	0.002
Neu^a^	4.5 ± 2.1	4.6 ± 2.4	4.3 ± 2.1	0.209
NLR^a^	3.4	3.4	3.8	0.240
PLR^c^	141.9	136.4	160.0	0.011
Treatment parameter (*n* = 5)				
IAT modalities				0.413
TACE	863 (50.47%)	214 (50.0%)	91 (45.5%)	
HAIC	847 (49.53%)	214 (50.0%)	109 (54.5%)	
IAT sessions, median	3	3	2	1.000
Combination with TKI				0.321
Absence	1170 (68.42%)	306 (71.5%)	144 (72.0%)	
Presence	540 (31.58%)	122 (28.5%)	56 (28.0%)	
Combination with ICI				0.277
Absence	1386 (81.05%)	360 (84.11%)	167 (83.5%)	
Presence	324 (18.95%)	68 (15.89%)	33 (16.5%)	
Sequential local therapy				0.721
Absence	1216 (71.11%)	298 (69.63%)	138 (69.0%)	
Presence	494 (28.89%)	130 (30.37%)	62 (31.0%)	
The response of first IAT				0.490
CR	31 (1.81%)	10 (2.34%)	6 (3.0%)	
PR	571 (33.39%)	143 (33.41%)	77 (38.5%)	
SD	917 (53.63%)	220 (51.4%)	94 (47.0%)	
PD	191 (11.17%)	55 (12.85%)	23 (11.5%)	

Abbreviations: *HCC* hepatocellular carcinoma, *HBV* hepatitis B virus, *HCV* hepatitis C virus, *ALT* alanine aminotransferase, *AST* aspartate aminotransferase, *AFP* α-fetoprotein, *γ-GT* gamma glutamyltransferase, *AKP* alkaline phosphatase, *ALB* albumin, *TBIL* total bilirubin, *BIL* bilirubin, *Cr* creatinine, *Glu* glucose, *CHE* cholinesterase, *HB* hemoglobin, *LYM* lymphocyte, *NEU* neutrophils, *PT* prothrombin time, *PTA* prothrombin activity, *INR* international normalized ratio

^a^Data are mean ± standard deviation

^b^Data are frequencies with percentages in parentheses

^c^Data are median value; *p* value < 0.05 indicated a significant difference

### Predictive performance comparison of the ML models

**Fig. 3:**
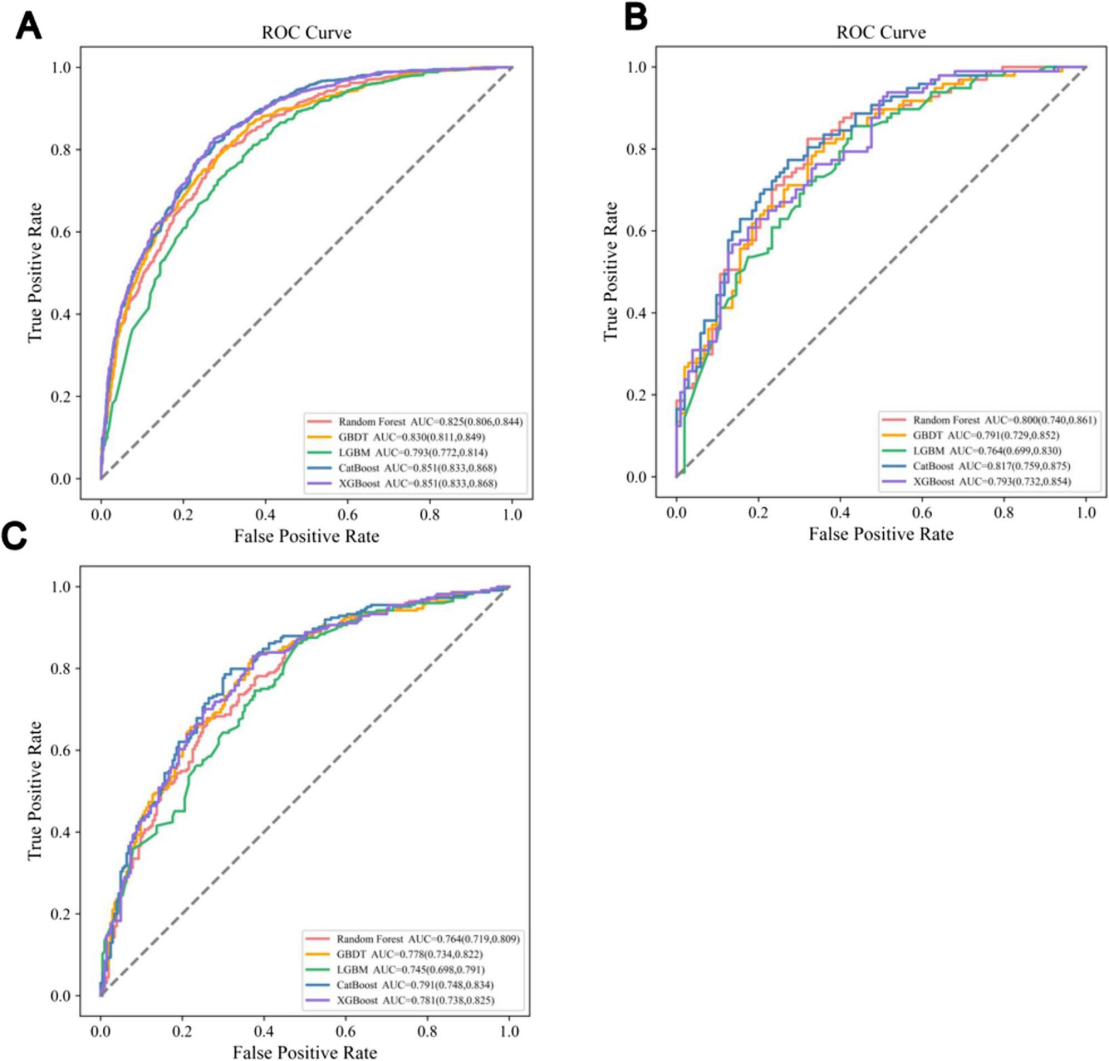
The performance comparison of five different ML models: **A** the AUC comparison in training datasets; **B** the AUC comparison in internal validation datasets; **C** the AUC comparison in external validation datasets

We selected five ML-based models, including XGBoost, CatBoost, RF, GBDT, and LGBM, for predictive performance comparison and testing. Figure 3A–C show the comparison of AUCs among five different ML models in three datasets, and the AUC, SENS, SPEC, PPV, and NPV of each model are shown in Table 2. The CatBoost model achieved the best prediction performance among all ML models. Herein, the CatBoost model trained had an AUC of 0.851 (95%CI, 0.833–0.868) for the TD, 0.817 (95%CI, 0.759–0.857) for the ITD, and 0.791 (95%CI, 0.748–0.834) for the ETD when trained using the 12 most important variables measured by the CatBoost importance score (Table 3). The feature importance of the CatBoost model and the most important features were as follows (in order of importance): response to the first IAT, ICI, maximum tumor size, BCLC grade, local therapy, PLT, PT, INR, CRP, TKI, AST, and Cre. In addition, we compared performance among the five ML models for the prediction of 1-, 2-, and 3-year death in three datasets (Table S4–S6). Interestingly, the predictive ability of the Catboost model remained superior to the other ML models.

**Table 2:** The performance comparison of five ML-based models

Models	Cohorts	AUC	NPV	PPV	SENS	SPEC	F1 score
CatBoost	TD	0.851	0.770	0.744	0.817	0.724	0.779
	ITD	0.791	0.743	0.740	0.786	0.696	0.762
	ETD	0.817	0.750	0.728	0.773	0.728	0.750
GBDT	TD	0.830	0.751	0.725	0.801	0.702	0.761
	ITD	0.778	0.731	0.710	0.821	0.632	0.762
	ETD	0.791	0.725	0.681	0.814	0.641	0.742
LGBM	TD	0.793	0.721	0.706	0.748	0.694	0.726
	ITD	0.745	0.699	0.663	0.862	0.520	0.750
	ETD	0.764	0.710	0.654	0.856	0.573	0.741
RF	TD	0.825	0.753	0.728	0.802	0.705	0.763
	ITD	0.764	0.708	0.742	0.679	0.740	0.709
	ETD	0.800	0.750	0.708	0.825	0.680	0.762
XGBoost	TD	0.851	0.775	0.747	0.827	0.725	0.785
	ITD	0.781	0.734	0.710	0.830	0.628	0.765
	ETD	0.793	0.720	0.753	0.629	0.806	0.685

Abbreviations: *TD* training datasets, *ITD* internal test datasets, *ETD* external test datasets, *RF* Random Forest, *GBDT* Gradient Boosting Decision Tree, *LGBM* Light GBM, *ML* machine learning, *AUC* areas under receiver operating characteristic curve, *PPV* positive predictive value, *NPV* negative predictive value, *SENS* sensitivity, *SPEC* specificity

**Table 3:** Variables selected using CatBoost and the corresponding variable importance score

Variables	Importance score
The response of first IAT	19.119
Combination with ICI	14.066
CRP	10.662
Maximum diameter of tumors	10.376
PLT	7.918
INR	6.574
BCLC grade	6.312
Sequential local therapy	6.057
PT	5.945
Combination with TKI	5.176
Cre	4.004
AST	3.791

Abbreviations: *IAT* intra-arterial therapy, *ICI* immune checkpoint inhibitors, *TKI* tyrosine kinase inhibitors, *CRP* C-reactive protein, *AST* aspartate aminotransferase, *Cre* creatinine, *PT* prothrombin time, *INR* international normalized ratio, *BCLC* Barcelona Clinic Liver Cancer

### Interpretation methods for the Catboost model

We used the SHAP method to explain the prediction results of the CatBoost model (Figure S1), and the nonlinear impact of the top 12 variables on OS is illustrated in Figures S2 to S13. Our results show that the PD of the first IAT had the highest risk for poor prognosis, and the risk for death was higher in SD compared with PR and CR, but the SHAP value was still less than 0 (Figure S2). Patients who received IAT alone had the second highest risk for the outcome of interest than those who received IAT combined with ICI (Figure S3). Patients with tumors > 10 cm and 5–10 cm and BCLC-B&C stage had a higher risk for poor prognosis than patients with tumors < 5 cm and BCLC-A stage (Figures S4–S5). Patients who received IAT alone had a much higher risk for the outcome than those who received IAT combined with sequential local therapy (Figure S6). The relationship between laboratory findings, including PLT, PT, INR, and CRP and SHAP value, was an S-shaped curve, with clear turning points (Figures S7–S10). The risk of death in HCC patients who received IAT increased significantly when certain examination variables, including PLT and CRP, and INR were present in elevated concentrations or PT value was decreased. The patients who received IAT combined with TKI had the least survival benefit among all combination schemes and had a lower risk for the outcome than those who received IAT alone (Figure S11). The relationship between AST and Cre and SHAP value was an S-shaped curve, with a decreasing trend (Figures S12–S13).

### RSSM construction and test

**Fig. 4:**
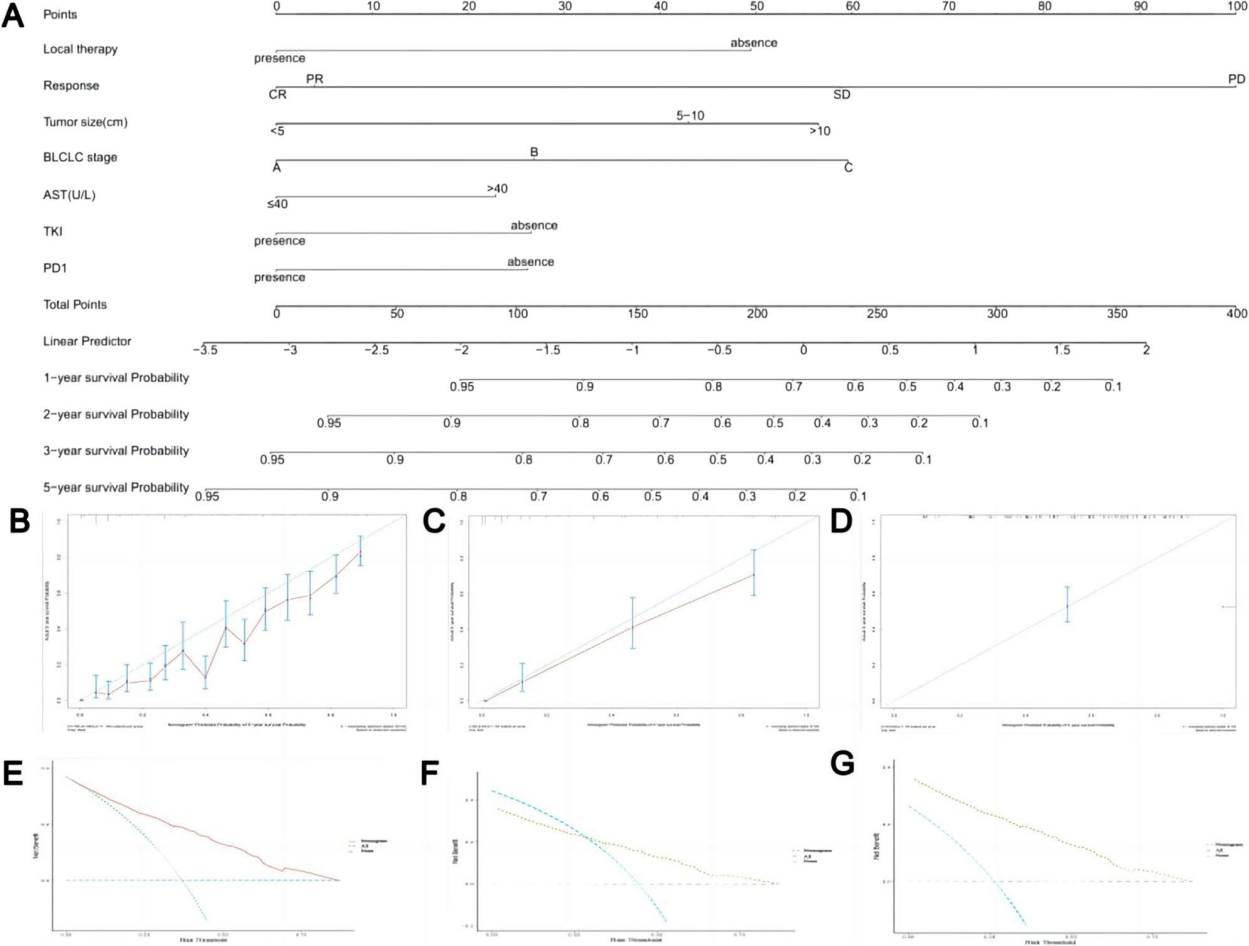
A RSSM with nomogram format was develop and validated: **A** the graph shows the nomogram for predicting 1-, 2-, 3-, and 5-year OS in HCC patients underwent IAT. **B**–**D** The bootstrapped calibration curves plotted with 1-, 3-, and 5-year OS were well matched with the idealized 45° line for the nomogram in the three datasets; **E**–**G** the decision curve analysis graphically indicated that the nomogram provided a larger benefit across the range of reasonable threshold probabilities in the three datasets

Stratifying prognoses for specific patient populations plays an important role in defining inclusion criteria for clinical trials and interventions. Thus, we constructed an RSSM using six variables, including ICI (HR, 0.678; 95%CI 0.549, 0.837), TKI (HR, 0.702; 95%CI 0.605, 0.814), local therapy (HR, 0.104; 95%CI 0.014, 0.747), response to the first IAT (HR, 4.221; 95%CI 2.229, 7.994), tumor size (HR, 1.054; 95%CI 1.038, 1.070), and BCLC grade (HR, 2.375; 95%CI 1.950, 2.894) based CatBoost model (Table S3). A nomogram was constructed to aid in the interpretation and visualization of this RSSM (Fig. 4A). The bootstrapped calibration curves plotted with 1-, 3-, and 5-year OS were well matched with the idealized 45° line for the nomogram in the three datasets (Fig. 4B–D). Decision curve analysis (DCA) graphically indicated that the nomogram provided a larger benefit across the range of reasonable threshold probabilities in the three datasets (Fig. 4E–G).

### Evaluation of the RSSM

**Fig. 5:**
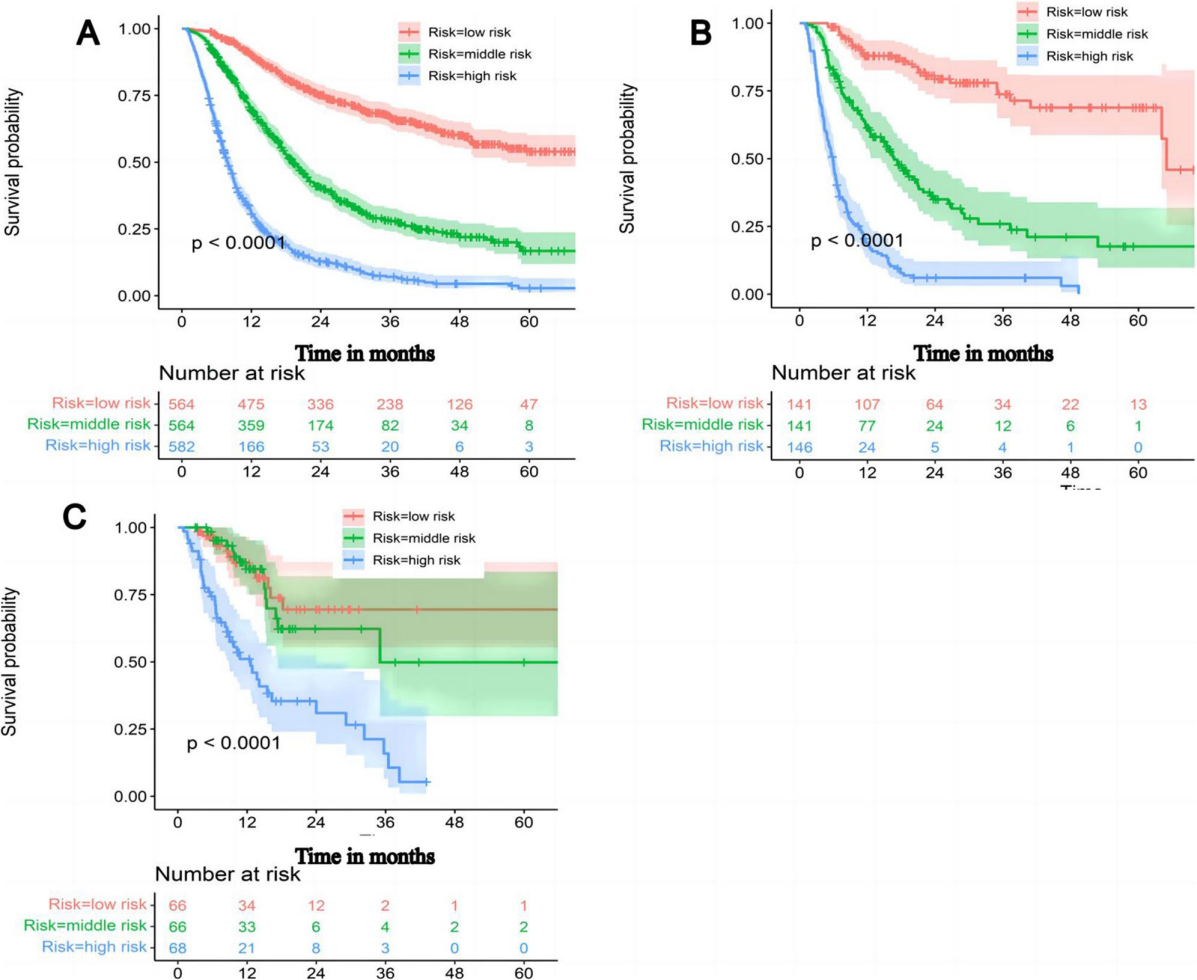
Overall survival (OS) of unresectable hepatocellular carcinoma patients are stratified based on RSSM. **A** The OS comparison between low-risk group, middle-risk group, and high-risk group in training datasets; **B** OS comparison between low-risk group, middle-risk group, and high-risk group in the internal validation datasets; **C** OS comparison between low-risk group, middle-risk group, and high-risk group in the external validation datasets

To facilitate the clinical practice of RSSM, we divided HCC patients into three groups according to the risk cutoff values (42.86 and 105.43), including high-risk, middle-risk, and low-risk groups. This pragmatic visualization of the risk level may help determine the IAT strategy for HCC patients. The 1-year, 3-year, and 5-year OS were 90.1%, 66.7%, and 54.0%, respectively, in the low-risk group, which was better than that in the middle-risk group (69.3%, 27.9%, and 16.7%, respectively) and high-risk group (30.6%, 7.1%, and 2.7%, respectively) in TD (*p* < 0.001) (Fig. 5A). Similarly, the cumulative 1-, 3-, and 5-year OS rates among the high-risk, middle-risk, and low-risk groups were also significantly different in the other two validation datasets (both, *p* < 0.001) (Fig. 5B, C). For convenient uptake in a clinical setting, a user-friendly online application of the RSSM was uploaded on our website (https://prehaicnomogramforhcc.shinyapps.io/DynNomapp/) (Fig S14).

## Discussion

IATs are promising options for treating unresectable HCC. Increasing evidence indicates that IAT treatment can significantly improve the survival benefits and quality of life of HCC patients [24–28]. Although an increasing number of models for outcome prediction after IATs have been built, a convenient and accurate tool that stratifies the prognosis risk of unresectable HCC remains lacking. Therefore, we decided to establish an RSMM to guide physicians in developing a comprehensive IAT scheme. The RSSM accurately stratifies patients who underwent IATs into three subgroups with significantly different cumulative long-term OS, which may potentially benefit personalized decision making and reduce future side effects and economic burden for more patients.

There are several strengths and novelties in this study. First, the RSSM was built and externally tested using a large cohort with a median follow-up time of 31.7 months from 12 hospitals. Different types of clinical variables, including demographic characteristics, tumor characteristics, laboratory tests, and treatment schemes, were used to construct ML models and ensure the comprehensiveness, robustness, and accuracy of these models from different perspectives. Second, we built five ML models and chose the optimal ML model to develop the RSSM for stratifying prognosis risk. CatBoost showed the optimal predictive ability with 12 top variables replacing 34 variables and can be accessed online conveniently. In addition, the RSSM could identify a specific HCC patient who received IAT with a homogeneous death risk. Of note, the RSSM has high predictive power with a visualized nomogram, assisting targeted trials and interventions. Third, although ML model was difficult to interpret, we used the SHAP method to explain the top 12 variables in the CatBoost model.

We identified 12 risk factors for prognosis outcome in the Catboost model and explained the relationship between these variables and death using the SHAP method [22]. The response to the first IAT plays an important role in the prognosis risk of HCC patients who underwent IAT (Figure S3). This result indicated that effective reduction in tumor burden is helpful for the long-term survival of HCC patients. Moreover, the results indicate that combination with ICI, TKI, and sequential local therapy contribute to survival benefit, which is worthy of attention from physicians in clinical practice (Figures S4, S7, and S11) [8, 9, 29]. Similar to previous studies, advanced HCC patients who received IAT combined with TKIs (e.g., lenvatinib and sorafenib) or PD-1 had better survival outcomes than those who received IATs alone [30–32]. Tumor diameter and BCLC stage were two important factors in the ranking of feature importance, which mainly reflected tumor characteristics defined by the guidelines. In clinical practice, HCC of various sizes and in different clinical stages also determines the formulation of treatment schemes (Figures S5, S6). Other laboratory test data variables included CRP, Cre, PT, INR, AST, and PLT. These indicators are closely related to the liver function status, coagulation mechanism, and the tumor microenvironment (TME) component assembly [33]. Among them, CRP is an acute phase protein and a well-accepted biomarker of cancer-induced systemic inflammation, which has also been directly linked to tumor progression. In the TME, inflammation has several tumor-promoting effects, including fostering cancer cell proliferation, metastatic seeding, and angiogenesis, as well as inhibition of adaptive immunity [33, 34]. Notably, IAT selection between TACE and HAIC did not change the survival outcome, which means that the selection of IAT modalities was not a determining factor for long-term survival.

The RSSM effectively divided HCC patients into subgroups based on different risk levels and helped distinguish optimal candidates for IAT. The high-risk group covering 33% of unresectable HCC patients in the TD had a 5-year death rate of 54.0%, whereas the middle- and low-risk groups had 5-year death rates of 16.7% and 2.7%, respectively. The RSSM is clinically relevant because it allowed the identification of a small but potentially manageable portion of patients who underwent IAT at high risk of death. For patients who underwent HAIC in the high-risk group based on the RSSM, we either changed the treatment scheme in advance or implemented a postoperative prevention and monitoring strategy. Although ICIs or TKIs combined with IATs or triple therapy have been proven to improve the survival benefit of advanced HCC, cases of HCC may still be heterogeneous with diverse outcomes. The RSSM comprised simple, readily available clinicopathological variables, which could identify HCC patients with high risk. High-risk patients should undergo intensive surveillance lasting as much as possible because of the high risk for death, accompanied by adjuvant systemic therapies.

Our study has several limitations. First, this study enrolled patients from several hospitals across our country, and the application scheme and duration of chemotherapy drugs are different at each study center. For example, the infusion time of fluorouracil in some hospitals was 23 h, while in others it was 46 h [35]. Moreover, there were also some differences in the selection of chemotherapy drugs. These above factors may affect the final outcome. Second, this study enrolled mostly patients with large HCC and HBV infection as a predominant etiology of HCC in China. It remains to be elucidated whether these results are widely applicable in Western countries, where the majority of patients have a low tumor burden or in which alcoholic liver cirrhosis is the predominant etiology. Third, information regarding complications during and after IAT, TKI, and ICI was not analyzed, warranting further investigation. Fourthly, additional multidimensional data derived from baseline imaging (radiomics analysis) might improve outcome prediction of IAT, and should be added to such models in future studies [36].

In conclusion, we have developed and externally validated a RSSM to predict prognosis after IATs in patients with unresectable HCC. The RSSM has been accessible online, which could be easily implemented in clinical practice for physicians and patients to stratify risk and predict prognosis quickly and accurately, thereby serving as a more favorable tool to strengthen individualized IAT in patients with unresectable HCC.

### Supplementary Information

Below is the link to the electronic supplementary material.

Supplementary file1 (PDF 688 KB)

## Abbreviations

CatBoost: Categorical Gradient Boosting
CI: Confidence intervals
GBDT: Gradient Boosting Decision Tree
HAIC: Hepatic arterial infusion chemotherapy
HCC: Hepatocellular carcinoma
HR: Hazard ratios
IATs: Intra-arterial therapies
ICI: Immune checkpoint inhibitors
LGBM: Light Gradient Boosting Machine
ML: Machine learning
OS: Overall survival
RF: Random Forest
RSSM: Risk scoring scale model
SHAP: SHapley Additive exPlanations
TACE: Transarterial chemoembolization
TKI: Tyrosine kinase inhibitors
XGBoost: EXtreme Gradient Boosting

## Declarations

### Guarantor

The scientific guarantor of this publication is Dr. Chengzhi Li.

### Statistics and biometry

No complex statistical methods were necessary for this paper.

### Study subjects or cohorts overlap

None of the study subjects or cohorts have been previously reported.

### Methodology

• retrospective

• diagnostic and prognostic study

• multicenter study
