# Supplementary material for: Prognosis prediction and risk stratification of transarterial chemoembolization or intraarterial chemotherapy for unresectable hepatocellular carcinoma based on machine learning
Source: Eur Radiol. 2024 Jan 30;34(8):5094–107. doi: 10.1007/s00330-024-10581-2 (PMC11255051; doi:10.1007/s00330-024-10581-2)
Supplement: Supplementary file 1 — Supplementary file1 (PDF 688 KB) [file 330_2024_10581_MOESM1_ESM.pdf]

Prognosis prediction and risk stratification of transarterial chemoembolization or intraarterial chemotherapy for unresectable hepatocellular carcinoma based on machine learning

Electronic Supplementary Material

1. Supplementary Information

E1.1 TACE or HAIC procedures

E1.2 The molecular-targeted agents and immune checkpoint inhibitors protocol

E1.3 IAT conversion therapy protocol

E1.4 The inclusion and exclusion criteria of enrolled patients

E1.5 Definitions of clinical variables

2. Supplementary Tables

Table S1. The data source form multi-center hospitals

Table S2. The details of the 38 variables in this study.

Table S3. The parameters of the five ML Algorithms

Table S4. Multivariable Regression Analysis for OS in training datasets

Table S5. Five ML based models for prediction of 1- year, 2- year and 3-year OS in training datasets

Table S6. Five ML based models for prediction of 1- year, 2- year and 3-year OS internal test datasets

Table S7. Five ML based models for prediction of 1- year, 2- year and 3-year OS in external test datasets

3. Supplementary Figures

Figure S1. The importance ranking of 12 variables in the CatBoost model using the SHAP algorithm.

Figure S2. shows the influence of the response to first IAT on 5-years death in the CatBoost model through the SHAP algorithm.

Figure S3. shows the influence of the ICI on 5-years death in the CatBoost model through the SHAP algorithm.

Figure S4. shows the influence of the tumor size on 5-years death in the CatBoost model through the SHAP algorithm.

Figure S5. shows the influence of the BCLC stages on 5-years death in the CatBoost model through the SHAP algorithm.

Figure S6. shows the influence of the local therapy on 5-years death in the CatBoost model through the SHAP algorithm.

Figure S7. shows the influence of the PLT on 5-years death in the CatBoost model through the SHAP algorithm.

Figure S8. shows the influence of the PT on 5-years death in the CatBoost model through the SHAP algorithm.

Figure S9. shows the influence of the INR on 5-years death in the CatBoost model through the SHAP algorithm.

Figure S10. shows the influence of the CRP on 5-years death in the CatBoost model through the SHAP algorithm.

Figure S11. shows the influence of the TKI on 5-years death in the CatBoost model through the SHAP algorithm.

Figure S12. shows the influence of the AST on 5-years death in the CatBoost model through the SHAP algorithm.

Figure S13. shows the influence of the Cre on 5-years death in the CatBoost model through the SHAP algorithm.

Figure S14. A web tool for prediction OS of HCC patients after IAT.

1. Supplementary Information

E1.1TACE or HAIC procedures

TACE or HAIC procedures have been described in our previous report [1-3]. All equipment of IAT procedures included i) digital subtraction angiography (Philips, type FD 20 1250 mA); ii) the artery sheath catheter was inserted into the femoral artery using the modified Seldinger technique; iii) A 5-Fr Yashiro catheter (Terumo) was advanced into the celiac trunk and superior mesenteric artery to assess the feeding hepatic artery; iv) A 2.7-Fr micro-catheter (Terumo) was inserted in the feeding artery. The therapeutic principles of IAT procedures were as follows: 1). TACE: the feeding artery was selected or super-selected whenever possible. Emulsion, which consisted of 10–20 ml lipiodol, 30–50 mg platinum drugs, and 20–40-mg epirubicin was injected slowly until the offending vessel occluded. If necessary, embolization using gel foam mixed with contrast medium was injected to reduce the residual blood flow until there was no longer any tumor staining after repeat angiography. 2). HAIC: all chemo-drugs were given by HAIC through the micro-catheter. A modified FOLFOX6 regimen, including oxaliplatin (130 mg/m^2 infusion for 3 h on day 1), leucovorin (200 mg/m^2 for 3–5 hours on day 1), and Fluorouracil (400 mg/m^2 in bolus, and then 2,400 mg/m^2 continuous infusion 23-46 h) was applied. Treatment was repeated every 3 weeks and commonly 4–6 cycles unless intrahepatic lesions progress or toxicity became unacceptable.

E1.2 The molecular-targeted agents and immune checkpoint inhibitors protocol

During HAIC or TACE treatment, molecular-targeted agents (MTAs) and immune checkpoint inhibitors (ICIs) were performed to control the intrahepatic and extrahepatic progression. Oral first-line targeted chemotherapy including sorafenib and lenvatinib was started 1–5 days after the first HAIC or TACE session and continually administered. Once the disease progresses or 3-4 AEs occur, the second-line treatment regimen (regorafenib or apatinib) can be administered. Oral lenvatinib (Lenvimafi;

Eisai Co., Ltd.) was administered to the patients with Ad-HCCs. The initial dose was determined based on the patient's body weight and liver function. Patients weighing > 60 kg with the Child–Pugh A classification started at a dose of 12 mg once daily. Patients weighing < 60 kg with the same liver function started at a dose of 8 mg once daily. A reduction in dosage or interruption of treatment was implemented when AEs were detected. Lenvatinib was administered unless patients were intolerant of radiological tumor progression or AEs. ICI immunotherapy were performed after 1-3 days IAT treat and every 3 weeks intravenously. Fixed-dose administration of PD-1 was used until disease progression or unexpected toxicity. The dose and interval of TKIs allowed changes depending on toxicity and disease conditions.

E1.3 IAT conversion therapy protocol

The target tumors were down-staged to BCLC-A stage and the tumor burden was reduced met the Milan criterion after multi-cycle of IAT treatment, the HCC patients were conducted by surgical resection, imaging-guided thermal ablation or SBRT. Among them, resectable tumor was defined as the complete removal of all macroscopic tumor tissue and an expected remnant liver volume no less than 250 mL/ M.

E1.4 The inclusion and exclusion criteria

The inclusion criteria were as follows: *(a)* age 18–75 years; *(b)* Eastern Cooperative Oncology Group (ECOG) performance status < 2; *(c)* Child-Pugh class A or B liver function; (d) the management of conventional TACE or HAIC of FOLFOX regimen. The exclusion criteria were as follows: *(a)* HCC combined with other malignancies; *(b)* patients underwent other treatment before IAT; *(c)* simultaneous treatment of TACE combined with HAIC; *(d)* the missing clinical data before IAT; *(e)* lost to follow-up > 12 months.

E1.5 Definitions of Variables

In this study, 38 clinical variables are collected as follows: (1) demographic and history variables (ECOG, pathology differentiation, weight，height, BMI, age, gender, comorbidities (i.e., hypertension, diabetes, heart disease, renal disease a esophageal gastric varices, etc.), etiology, CTP class, ALBI grade, ascites); (2) tumor features (maximal tumor diameter, number of tumor, tumor burden, macroscopic vascular invasion, metastasis, and BCLC stage); (3) laboratory findings (α-fetoprotein [AFP], des-γ-carboxy prothrombin [DCP], serum albumin; [ALB], total bilirubin [TB], platelet counts, prothrombin time (PT), international normalized ratio [INR], aspartate aminotransferase [AST] and alanine aminotransferase [ALT]), C reactive protein (CRP), creatinine, neutrophils, lymphocyte. Albumin- bilirubin (ALBI) grades were used to replace CTP grade for their objectiveness. ALBI score was calculated before treatment using the appropriate clinical parameters and ALBI grade was defined as follows: (log 10 bilirubin [BI] [μ mol /L] × 0.66) + (albumin [AL] [g/L] ×-0.085), (grade 1, 2, and 3 = ≤ -2.60, > -2.60 to -1.39, and > -1.39, respectively). For more detailed evaluations of patients with the middle grade of ALBI (grade 2), we used modified ALBI (mALBI) grading consisting of 4 levels, which included subgrading for the middle grade of 2 (2a and 2b) based on an ALBI score of −2.27 as the cut-off, which was previously reported as the value for indocyanine green retention after 15 min (ICG-R15) of 30% [4]; (4) treatment parameters (IAT modalities, combination with TKI, combination with ICI, sequential local therapy and the response of first IAT). The responses to IAT was assessed by dynamic contrast enhanced CT or magnetic resonance imaging (MRI) based on modified Response Evaluation Criteria in Solid Tumor (mRECIST), including complete response (CR), partial response (PR), stable disease (SD), and progression disease (PD), which was performed every 4–6 weeks after initial IAT and evaluated independently by two radiologists (reader 1, L.Z.L., and reader 2, J. Z., with 10 years of experience) who were blinded to IAT procedures at the time of data collection.

References

1 Ueshima K, Komemushi A, Aramaki T, et al (2022) Clinical Practice Guidelines for Hepatic Arterial Infusion Chemotherapy with a Port System Proposed by the Japanese Society of Interventional Radiology and Japanese Society of Implantable Port Assisted Treatment. Liver Cancer. 11(5):407-425. doi:10.1159/000524893

2 Yamasaki T, Saeki I, Yamauchi Y, et al (2022) Management of Systemic Therapies and Hepatic Arterial Infusion Chemotherapy in Patients with Advanced Hepatocellular Carcinoma Based on Sarcopenia Assessment. Liver Cancer. 11(4):329-340. doi:10.1159/000522389

3 Wang T, Dong J, Zhang Y, et al (2022) Efficacy and safety of hepatic artery infusion chemotherapy with mFOLFOX in primary liver cancer patients with hyperbilirubinemia and ineffective drainage: a retrospective cohort study. Ann Transl Med. 10(7):411. doi:10.21037/atm-22-9784.

4 Hiraoka A, Kumada T, Tsuji K, et al (2019) Validation of Modified ALBI Grade for More Detailed Assessment of Hepatic Function in Hepatocellular Carcinoma Patients: A Multicenter Analysis. Liver Cancer. 8(2):121-129. doi:10.1159/000488778

2. Supplementary Tables

Table S1. The data source form multi-center hospitals

**Hospitals' name**	**HAIC group (patients' number)**	**TACE group (patients' number)**
**Total**	**1170**	**1168**
Sun Yat-sen University Cancer Center	960	513
The First Affiliated Hospital of Sun Yat-sen University	75	112
The Third Affiliated Hospital of Sun Yat-sen University	56	130
Guangdong Provincial People's Hospital	36	60
Jinan University First Affiliated Hospital	23	59
Guangzhou Cancer Hospital	10	62
Affiliated Hospital of Southern Medical University	0	24
The First Affiliated Hospital of Nanchang University	0	25
The First Affiliated Hospital of Peking University	0	13
Affiliated Cancer Hospital of Chinese Academy of Medical Sciences	8	132
Luhe Hospital, Capital Medical University	2	38
Chinese PLA General Hospital	0	17

Table S2. The details of the 38 variables in this study.

Variable	Variable name	Abbreviation
**Demographic and history**		
	Age	
	Gender	
	Weight	
	Height	
	BMI	
	Pathology differentiation	
	ECOG	
	Etiology	
	Comorbidity score	CS
	AIBI grade	
	Child-Pugh grade	CTP grade
	Ascites	
**Tumor data**		
	Number of tumors	No. of tumors
	Maximum diameter of tumor	MD of tumor
	Tumor burden	TB
	BCLC grade	
	Vascular invasion	
	Metastasis	
**Treatment parameters**		
	IAT modalities	IATM
	TKI	TKI
	ICI	ICI
	Sequential local therapy	SLT
	The response of first IAT	TRFI
**Laboratory findings**		
	Alanine aminotransferase	ALT
	Aspartate aminotransferase	AST
	α-fetoprotein	AFP
	des-γ-carboxy prothrombin	DCP
	Albumin	ALB
	Total bilirubin	TBIL
	Creatinine	Cre
	C reactive protein	CRP
	Platelet	PLT
	Lymphocyte	LYM
	Neutrophils	NEU
	Neutrophils to lymphocyte	NLR
	Lymphocyte to lymphocyte	PLR
	Prothrombin time	PT
	Prothrombin activity	PTA
	International Normalized Ratio	INR

Table S3. The parameters of the ML Algorithms

No.	Algorithms	Details
1	RF	Random Forest Classifier (max_depth=6,max_features='sqrt',max_leaf_nodes=102,min_samples_leaf=22,min_samples_split=9,n_estimators=79,random_state=0)
2	GBDT	GradientBoostingClassifier(max_depth=2,min_samples_split=0.06,learning_rate= 0.1)
3	LGBM	LGBMClassifier(max_depth=4,min_child_samples=5,min_data_in_leaf=35,learning_rate= 0.01)
4	CatBoost	CatBoostClassifier(verbose=False,max_depth=6,subsample=0.868421052631579,bagging_temperature=0.5,n_estimators=173,learning_rate= 0.035789473684210524)
5	XGBoost	XGBClassifier(max_depth=3,min_child_weight=3,gamma=0.87,colsample_bytree=1.0,subsample=0.92,reg_alpha=0.5,reg_lambda=14,n_estimators=98,learning_rate=0.1)

Table S4 Multivariable Regression Analysis for OS in the training datasets

Variables	β	Hazard Ratio (95% CI)	P value
Response to the first IAT (SD+PD)	1.440	4.220 (3.410, 5.220)	<0.001
ICI (Absence)	2.324	0.678 (0.549, 0.837)	<0.001
TKI (Absence)	1.306	0.702 (0.605, 0.814)	<0.001
Local therapy (Absence)	2.657	0.104 (0.014, 0.747)	0.024
BCLC stage (C)	4.392	2.375 (1.950, 2.894)	<0.001
Tumor size (>10 cm)	2.781	1.054 (1.038, 1.070)	<0.001

Abbreviation. OS, overall survival; IAT, intra-arterial therapy; ICI, immune checkpoint inhibitors; TKI, tyrosine kinase inhibitors; BCLC, Barcelona Clinic Liver Cancer.

Table S5. Five ML based models for prediction of 1- year, 2- year and 3-year OS in training datasets

Models	Cohorts	AUC (95% CI)	ACC	SENS	SPEC	PPV	NPV
XGBoost	**1 year**	0.8228 (0.7885-0.8571)	0.7541	0.7457	0.7603	0.6948	0.8033
	**2 year**	0.8346 (0.8004-0.8688)	0.7723	0.7393	0.813	0.8296	0.7168
	**3 year**	0.7916 (0.7532-0.8299)	0.7304	0.7006	0.7733	0.8165	0.6421
CatBoost	**1 year**	0.8525 (0.8213-0.8837)	0.7851	0.8319	0.7508	0.7096	0.8592
	**2 year**	0.8808 (0.8528-0.9088)	0.8142	0.8218	0.8049	0.8384	0.7857
	**3 year**	0.8161 (0.7794-0.8528)	0.7523	0.7284	0.7867	0.831	0.6679
LGBM	**1 year**	0.8096(0.7738-0.8455)	0.7505	0.8319	0.6909	0.6632	0.8488
	**2 year**	0.8159(0.7794-0.8523)	0.7650	0.7822	0.7439	0.79	0.7349
	**3 year**	0.7917(0.7529-0.8305)	0.7322	0.713	0.76	0.8105	0.6477
RF	**1 year**	0.8355 (0.8026-0.8684)	0.7723	0.806	0.7476	0.7004	0.8404
	**2 year**	0.8387 (0.8055-0.872)	0.776	0.7657	0.7886	0.8169	0.7321
	**3 year**	0.8286 (0.7944-0.8628)	0.7468	0.7037	0.8089	0.8413	0.6547
GBDT	**1 year**	0.8467 (0.8143-0.879)	0.7851	0.7802	0.7886	0.7298	0.8306
	**2 year**	0.8363 (0.802-0.8706)	0.7869	0.8218	0.7439	0.7981	0.7722
	**3 year**	0.8268 (0.7914-0.8622)	0.7668	0.7469	0.7956	0.8403	0.6858

Abbreviation: AUC, areas under receiver operating characteristic curve ; ACC, accuracy ; PPV, positive predictive value ; NPV, negative predictive value; SENS, sensitivity; SPEC, specificity

Table S6. Five ML based models for prediction of 1- year, 2- year and 3-year OS in internal test datasets

Models	Cohorts	AUC (95% CI)	ACC	SENS	SPEC	PPV	NPV
XGBoost	**1 year**	0.8068 (0.7449-0.8688)	0.7377	0.9221	0.6038	0.6283	0.9143
	**2 year**	0.7304 (0.6557-0.805)	0.7158	0.8218	0.5854	0.7094	0.7273
	**3 year**	0.7149 (0.6404-0.7894)	0.6503	0.537	0.8133	0.8056	0.5495
CatBoost	**1 year**	0.8116 (0.7503-0.8729)	0.7158	0.9091	0.5755	0.6087	0.8971
	**2 year**	0.7443 (0.6704-0.8181)	0.7322	0.8317	0.6098	0.7241	0.7463
	**3 year**	0.7258 (0.6525-0.7991)	0.6940	0.7315	0.64	0.7453	0.6234
LGBM	**1 year**	0.8049 (0.7434-0.8665)	0.7322	0.9091	0.6038	0.625	0.9014
	**2 year**	0.7399 (0.6659-0.814)	0.7322	0.8218	0.622	0.7281	0.7391
	**3 year**	0.7190 (0.6445-0.7935)	0.6885	0.7315	0.6267	0.7383	0.6184
RF	**1 year**	0.8103(0.7485-0.8722)	0.7486	0.8442	0.6792	0.6566	0.8571
	**2 year**	0.7555(0.6827-0.8282)	0.7377	0.802	0.6585	0.7431	0.7297
	**3 year**	0.7211 (0.6437-0.7985)	0.7377	0.8333	0.6	0.75	0.7143
GBDT	**1 year**	0.7976 (0.7352-0.86)	0.7104	0.9351	0.5472	0.6	0.9206
	**2 year**	0.7446(0.6714-0.8178)	0.7268	0.7525	0.6951	0.7525	0.6951
	**3 year**	0.7285(0.6537-0.8033)	0.7268	0.75	0.6933	0.7788	0.6582

Abbreviation: AUC, areas under receiver operating characteristic curve ; ACC, accuracy ; PPV, positive predictive value ; NPV, negative predictive value; SENS, sensitivity; SPEC, specifity.

Table S7. Five ML based models for prediction of 1- year, 2- year and 3-year OS in external test datasets

Models	Cohorts	AUC (95% CI)	ACC	SENS	SPEC	PPV	NPV
XGBoost	**1 year**	0.8030 (0.7482-0.8577)	0.7510	0.7130	0.7836	0.7387	0.7609
	**2 year**	0.7402 (0.676-0.8056)	0.6827	0.6491	0.7564	0.8538	0.4958
	**3 year**	0.7048(0.6301-0.7795)	0.6345	0.5938	0.7719	0.8976	0.3607
CatBoost	**1 year**	0.8082(0.7536-0.8629)	0.7470	0.7217	0.7687	0.7281	0.763
	**2 year**	0.7403(0.6762-0.8044)	0.6546	0.5789	0.8205	0.8761	0.4706
	**3 year**	0.7021(0.6268-0.7774)	0.6546	0.6198	0.7719	0.9015	0.3761
LGBM	**1 year**	0.7953(0.7394-0.8511)	0.7631	0.5739	0.9254	0.8684	0.7168
	**2 year**	0.7233(0.6583-0.7884)	0.6867	0.6901	0.6795	0.8252	0.5
	**3 year**	0.6664 (0.5918-0.741)	0.6546	0.6562	0.6491	0.863	0.3592
RF	**1 year**	0.8006 (0.7448-0.8564)	0.7550	0.7913	0.7239	0.7109	0.8017
	**2 year**	0.7256 (0.6597-0.7915)	0.6747	0.6784	0.6667	0.8169	0.486
	**3 year**	0.6749 (0.5988-0.7509)	0.5020	0.3854	0.8947	0.9250	0.3018
GBDT	**1 year**	0.8005 (0.745-0.8559)	0.7550	0.6261	0.8657	0.8000	0.7296
	**2 year**	0.7242 (0.659-0.7893)	0.6345	0.5263	0.8718	0.9000	0.4564
	**3 year**	0.6789 (0.6054-0.7524)	0.6546	0.6510	0.6667	0.8681	0.3619

Abbreviation: AUC, areas under receiver operating characteristic curve ; ACC, accuracy ; PPV, positive predictive value ; NPV, negative predictive value; SENS, sensitivity ; SPEC, specificity.

3. Supplementary Figures

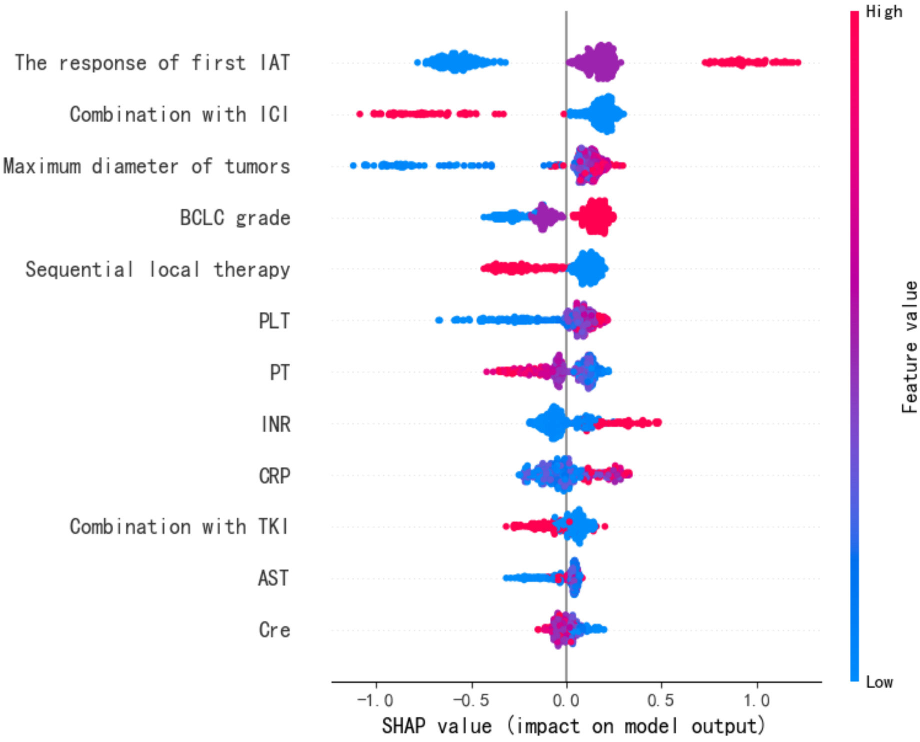

Figure S1. The importance ranking of 12 variables in the CatBoost model using the SHAP algorithm.

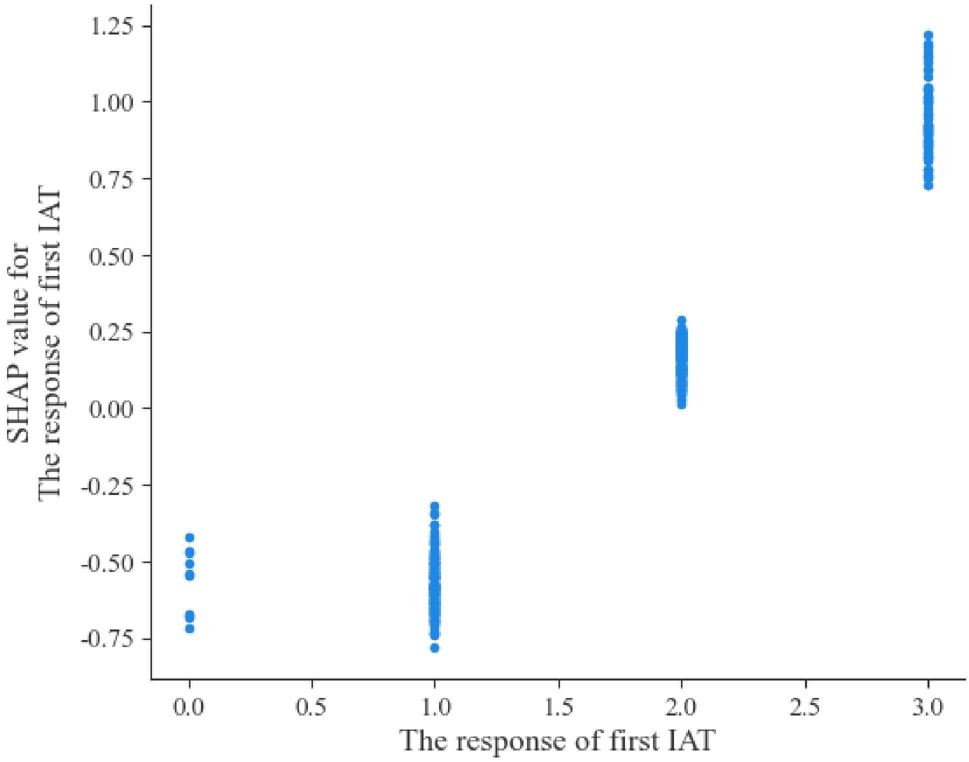

Figure S2. shows the influence of the response to first IAT on 5-years death in the CatBoost model through the SHAP algorithm.

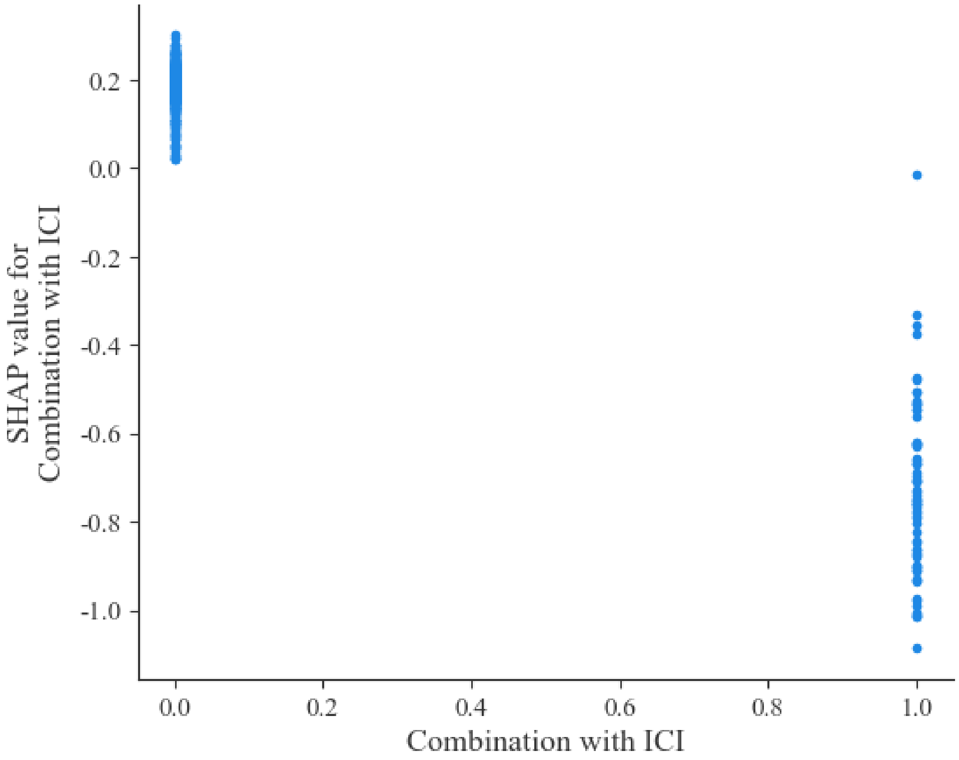

Figure S3. shows the influence of the ICI on 5-years death in the CatBoost model through the SHAP algorithm.

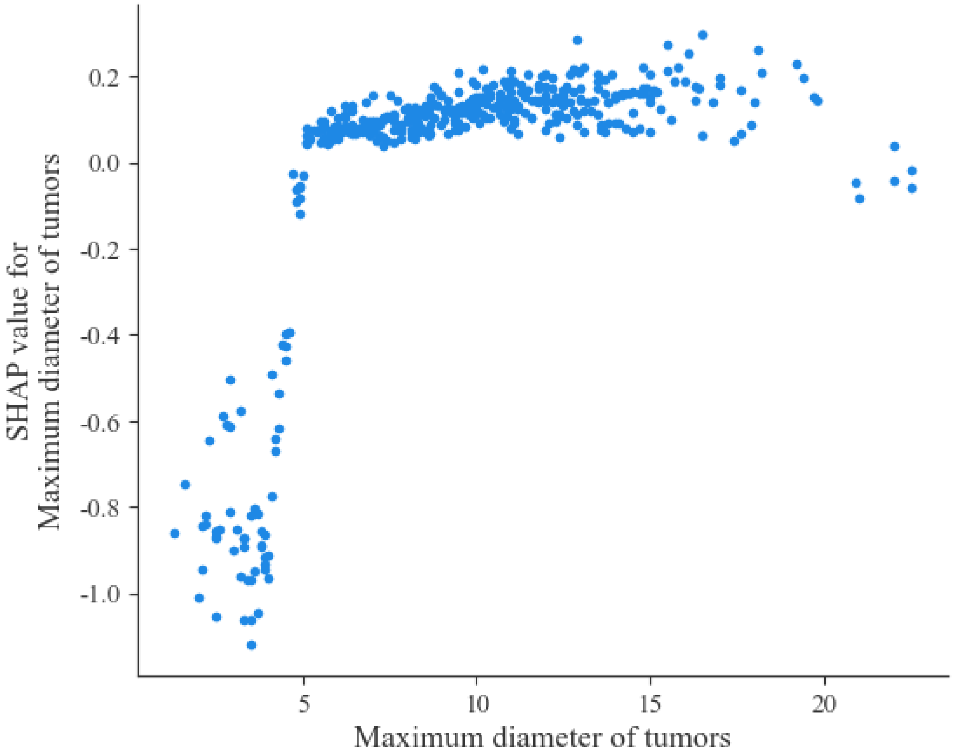

Figure S4. shows the influence of the tumor size on 5-years death in the CatBoost model through the SHAP algorithm.

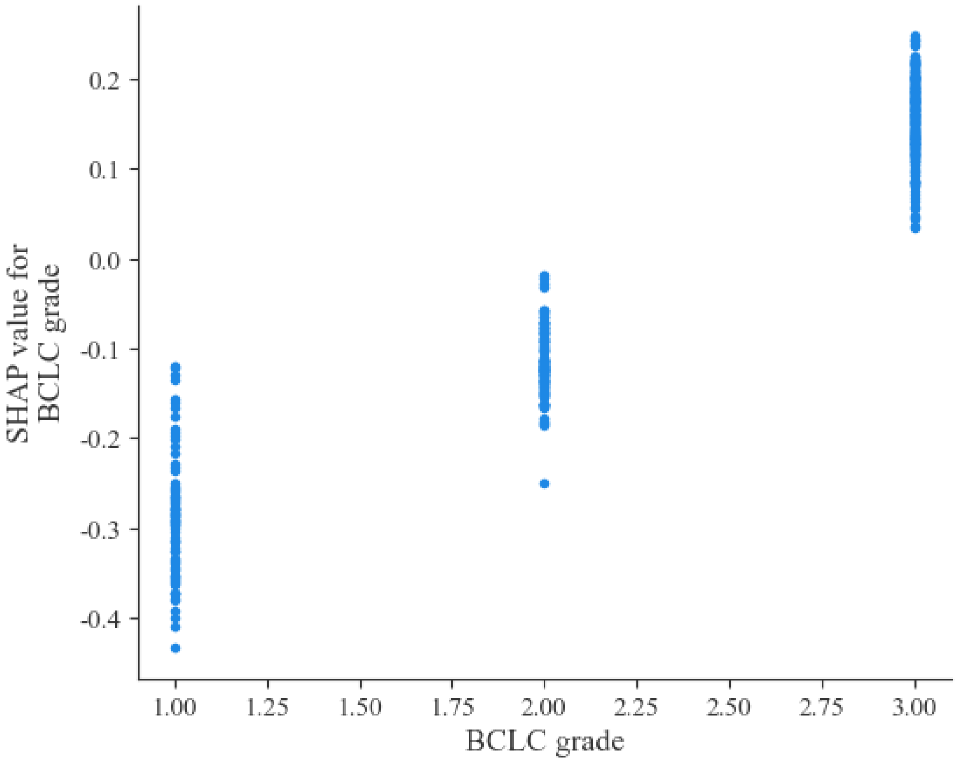

Figure S5. shows the influence of the BCLC stages on 5-years death in the CatBoost model through the SHAP algorithm.

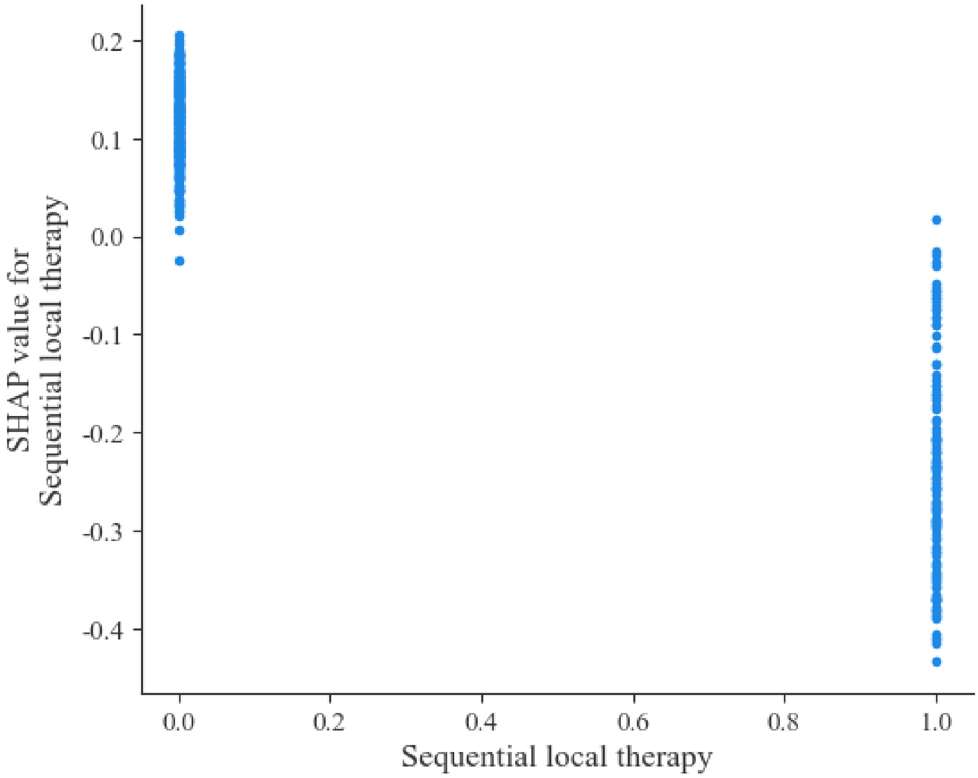

Figure S6. shows the influence of the local therapy on 5-years death in the CatBoost model through the SHAP algorithm.

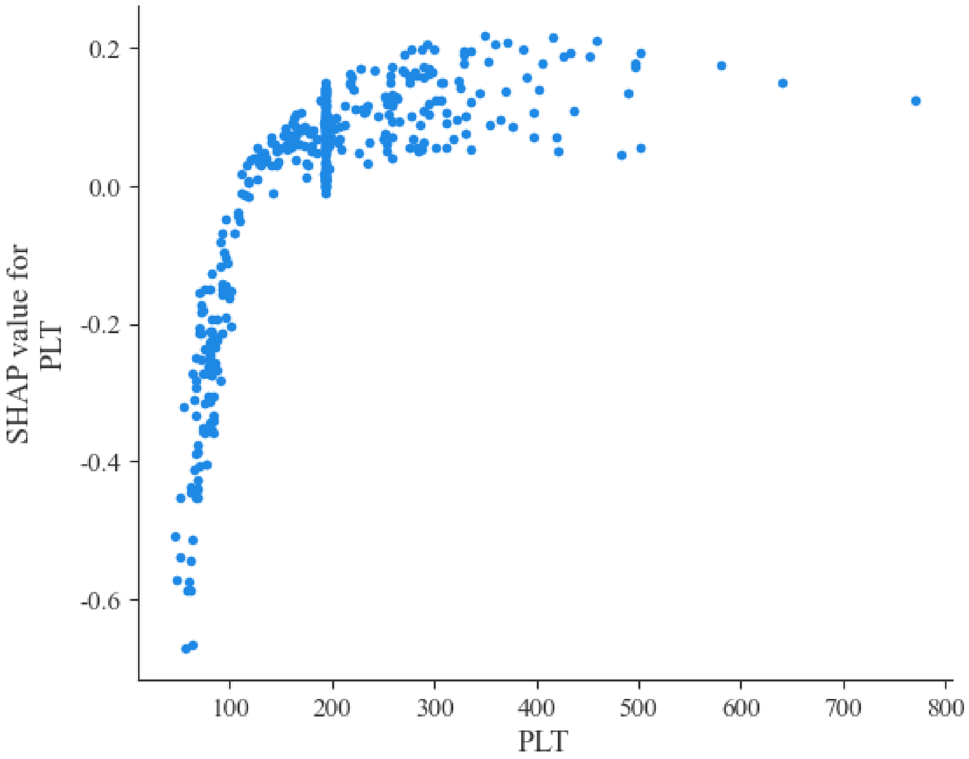

Figure S7. shows the influence of the PLT on 5-years death in the CatBoost model through the SHAP algorithm.

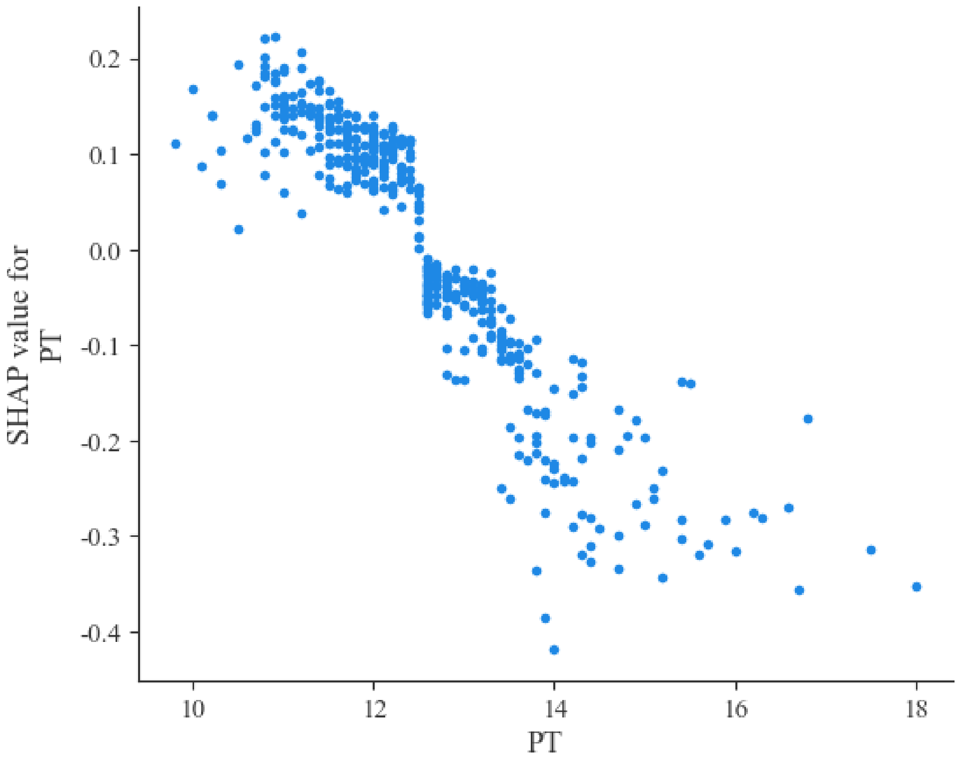

Figure S8. shows the influence of the PT on 5-years death in the CatBoost model through the SHAP algorithm.

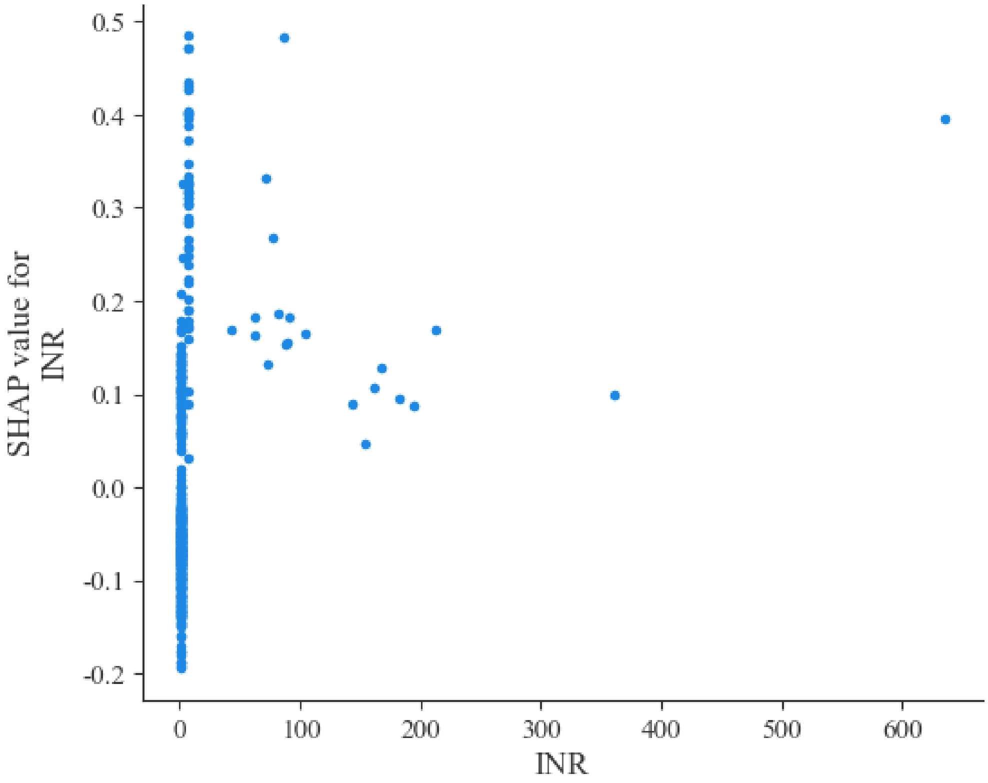

Figure S9. shows the influence of the INR on 5-years death in the CatBoost model through the SHAP algorithm.

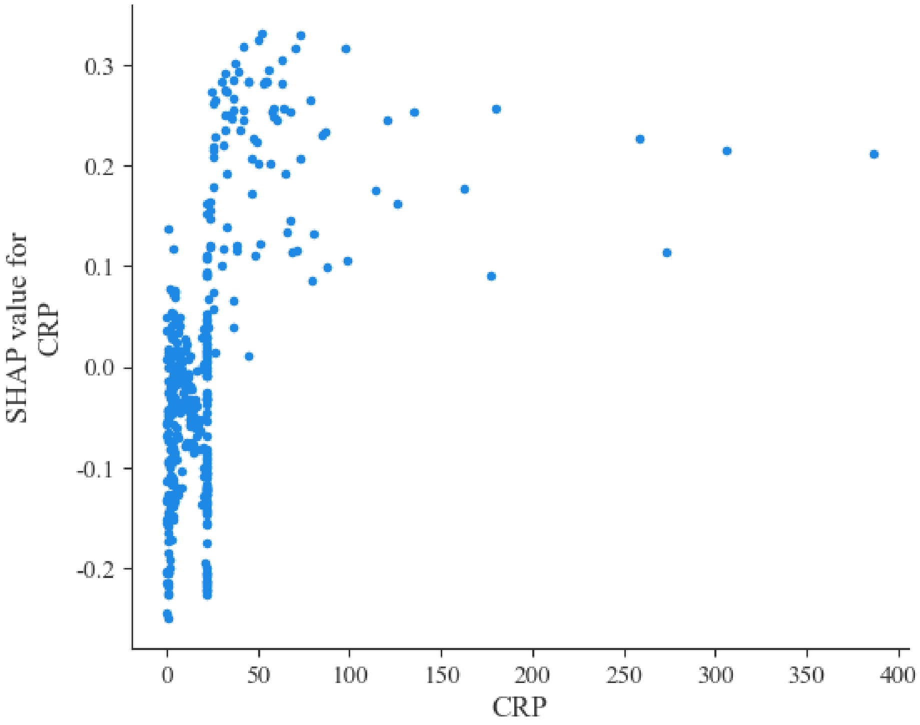

Figure S10. shows the influence of the CRP on 5-years death in the CatBoost model through the SHAP algorithm.

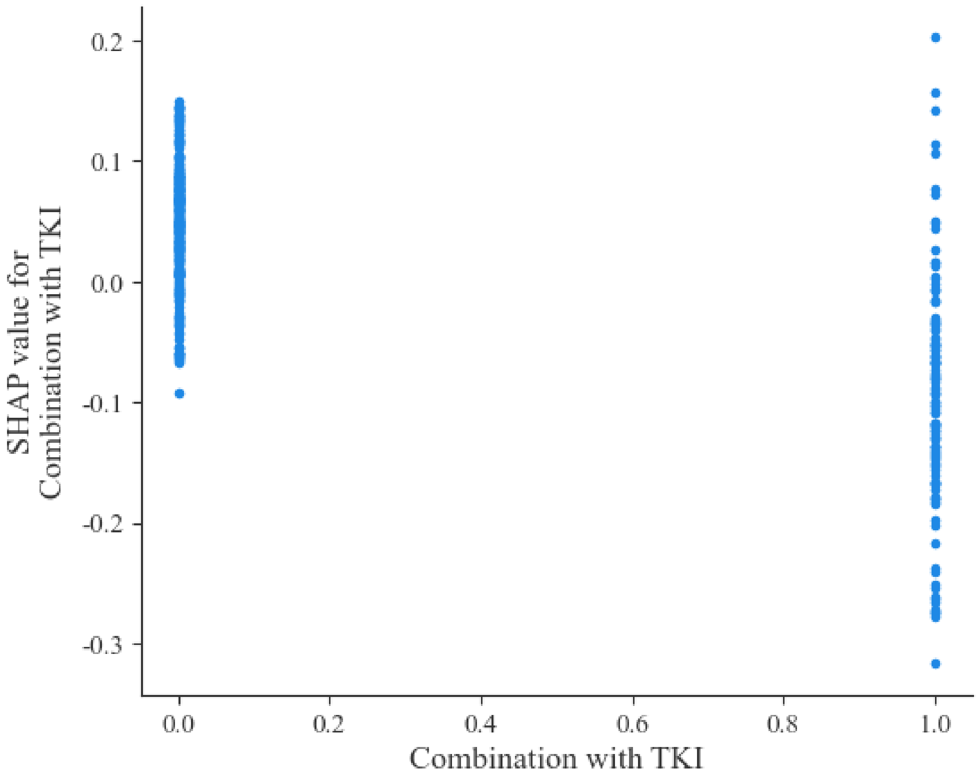

Figure S11. shows the influence of the TKI on 5-years death in the CatBoost model through the SHAP algorithm.

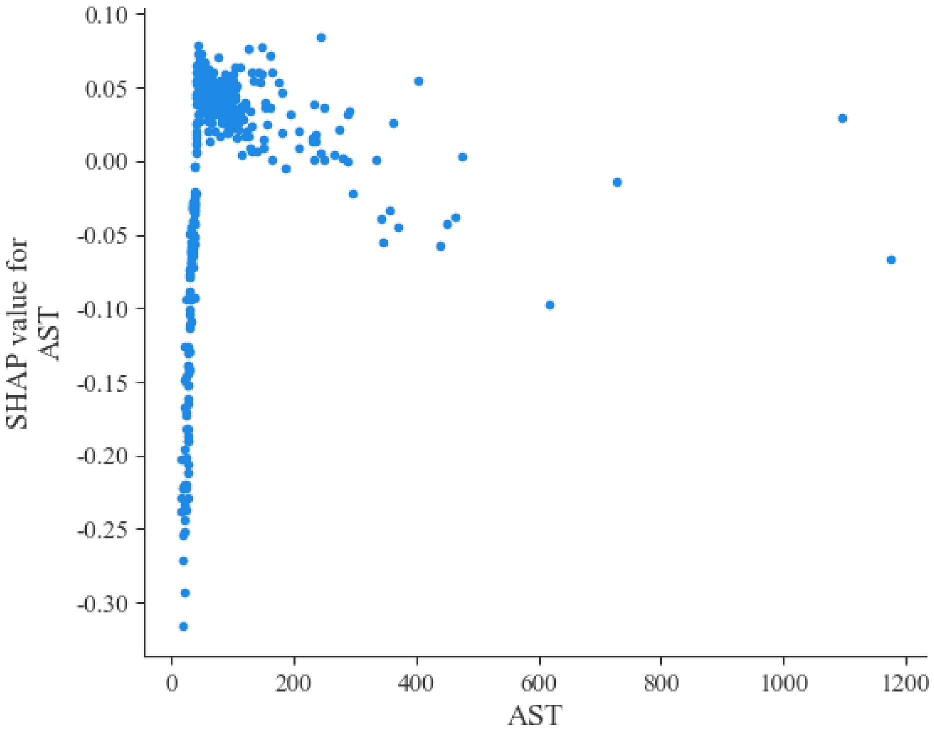

Figure S12. shows the influence of the AST on 5-years death in the CatBoost model through the

SHAP algorithm.

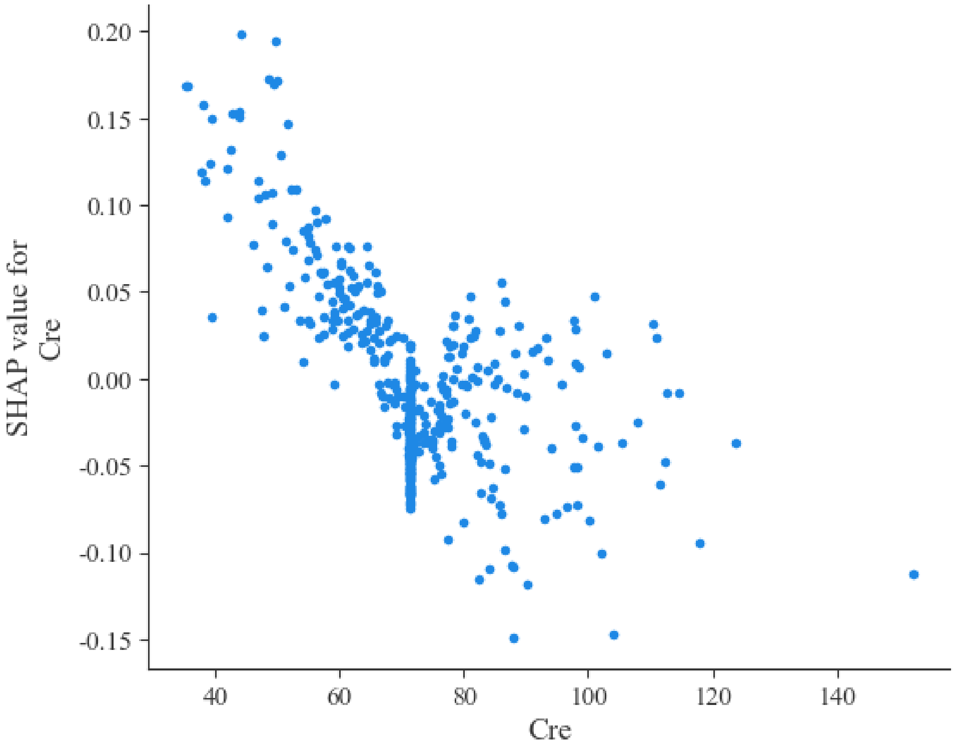

Figure S13. shows the influence of the Cre on 5-years death in the CatBoost model through the SHAP algorithm.

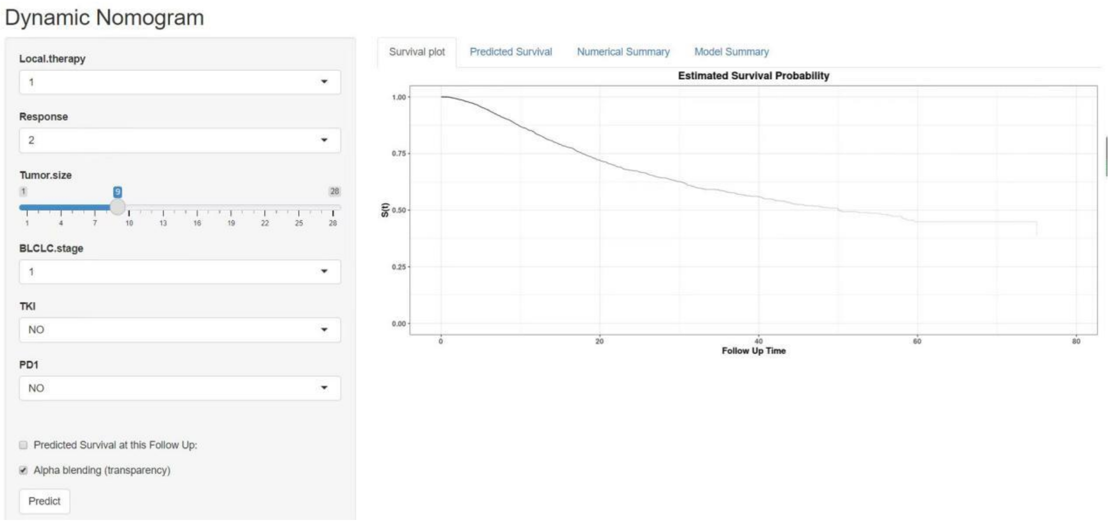

Figure S14. A web tool for prediction OS of HCC patients after IAT.
